# Investigation of micellar and interfacial phenomenon of amitriptyline hydrochloride with cationic ester-bonded gemini surfactant mixture in different solvent media

**DOI:** 10.1371/journal.pone.0241300

**Published:** 2020-11-06

**Authors:** Malik Abdul Rub

**Affiliations:** Chemistry Department, Faculty of Science, King Abdulaziz University, Jeddah, Saudi Arabia; Universidade de Aveiro, PORTUGAL

## Abstract

Herein, the interaction among the antidepressant drug amitriptyline hydrochloride (AMT) and a green gemini surfactant, ethane-1, 2-diyl bis(N,N-dimethyl-N-tetradecylammoniumacetoxy) dichloride (14-E2-14), via numerous techniques such as tensiometry, fluorimetry, FT-IR and UV-visible spectroscopy in three different media (aqueous 0.050 mol·kg^-1^ NaCl, 0.50 and 1.0 mol·kg^-1^ urea) were investigated. AMT is used to treat mental illness or mood problems, such as depression. The aggregation of biologically active ingredients can enhance the bioavailability of hydrophobic drugs. A significant interaction between AMT and 14-E2-14 was detected by tensiometric study as the critical micelle concentration (*cmc*) of AMT+14-E2-14 is reduced upon an increase of mole fraction (*α*_1_) of 14-E2-14. The decrease in *cmc* indicates the nonideality of studied mixtures of different compositions. Although, employed drug AMT is freely soluble in the aqueous and non-aqueous system but is not hydrophobic enough to act as its carrier. Instead, gemini surfactant formed spherical micelles in an aqueous system and their high solubilization capability, as well as their relatively lower cmc value, makes them highly stable in vivo. The *cmc* values of AMT+14-E-14 mixtures in all cases were further decreased and increased in NaCl and urea solutions respectively as compared with the aqueous system. Numerous micellar, interfacial, and thermodynamic parameters have been measured by applying various theoretical models. The obtained changes in the physicochemical assets of AMT upon adding of 14-E2-14 are likely to enhance the industrial and pharmaceutical applications of gemini surfactants. The negative interaction parameters (*β*^m^ and *β*^σ^), indicate synergistic attraction is occurring in the mixed systems. The aggregation number (*N*_agg_), Stern–Volmer constant (*K*_sv_), etc. are attained through the fluorescence method, also supporting the attractive interaction behavior of AMT+14-E2-14 mixtures in all solvents. The *N*_agg_ was found to increase in the salt solution and decrease in the urea system compared with the aqueous solution. FT-IR and UV-visible analysis also depict the interaction between the constituent alike tensiometry and fluorimetry methods. The results suggested that gemini surfactants may serve as a capable drug delivery agent for antidepressants, improving their bioavailability.

## 1. Introduction

The surfactant molecules enclose polar hydrophilic groups and nonpolar hydrophobic groups in a single molecule; therefore, surfactants are also called amphiphilic molecules [1, 2]. The exceptional composition of surfactants is the reason for their remarkable interaction abilities [1, 2]. Understanding the self-association features of surfactants is of immense interest because of their utilization in a broad range of appliances in both academic and industrial research [1, 2]. Nearly all surfactants self-assemble into micelles in aqueous and other solvents after reaching or surpassing a concentration known as the critical micelle concentration (*cmc*) [3–5]. The surfactant micelles in pharmaceutical sciences are of pivotal importance because of their capacity to solubilize and transport hydrophobic drugs using their palisade layers [6, 7]. Surfactants work as a drug carrier via some additives, and so comprehensive studies of the effects of numerous additives on the associated behavior of the drug are needed [7].

Gemini, or dimeric, surfactants are a special class of surfactants as they contain two amphiphilic fractions linked at or near the headgroups via spacers of diverse nature such as methylene, oxyethylene, or ester [2, 8]. At present, these surfactants are gaining attention as good solubilizers for various organic hydrophobic molecules. Due to their unique structure and properties, gemini surfactants more effective than conventional surfactants at, for example, lowering the surface tension and lowering *cmc* [9]. Gemini surfactants could conceivably be employed as capping agents during the synthesis of nanoparticles, drug carriers, antimicrobial compounds, microemulsions, or prototypes for the preparation of mesoporous ingredients [10, 11]. Furthermore, it is as well employed to compose DNA carrier fragments, which are adequate to transport genes towards cells of practically at all type DNA molecules in respect of size [12]. In the current study, a special gemini surfactant was used that contains a cleavable ester bonded spacer which is found in nature [13, 14]. The ester bonded spacer of gemini is highly polar in nature; therefore, it pays higher aqueous solubility that making them simply degradable [13, 14]. The biodegradable gemini surfactants formed micelles at very low concentrations, which indicated that *cmc* values were lower than those of conventional surfactants, suggesting that biodegradable gemini surfactants may be more suitable for the solubilization of hydrophobic compounds than conventional surfactants [15, 16].

Like surfactants, there has been an expansion of studies in current years exploring the self-association potential of amphiphilic drugs, which have surface activity akin to typical surfactants [7, 17–19]. Compared to the self-aggregation of single amphiphile systems, amphiphile-additive mixed systems form mixed aggregates with great efficiency and at a low cost. The resulting mixed micelles have desirable high surface activity [20]. A mixed system can demonstrate better interfacial property along with diverse colloidal assets from either of the constituents. As a result, in pharmaceutical sciences, mixed micelle systems are employed to increase the absorption of a range of drugs in humans [7].

**Scheme 1:**
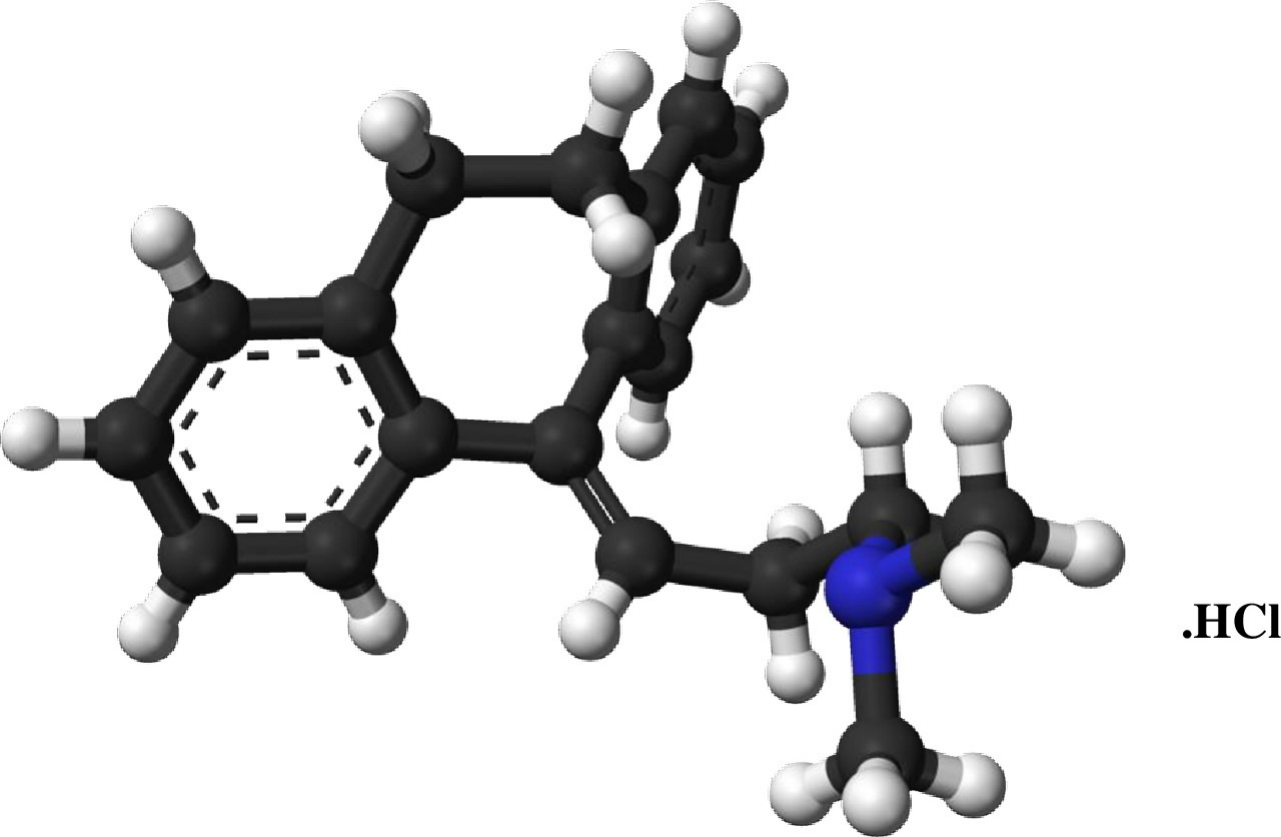
Molecular structure of amitriptyline hydrochloride (AMT).

Amitriptyline hydrochloride (AMT) is an amphiphilic drug, used as an antidepressant. AMT has a rigid, nearly planar, tricyclic ring structure linked through a small hydrocarbon chain with a terminal N-group. Their molecular structure is shown in Scheme 1 [7, 21]. This class of drugs usually forms micelles at higher concentrations compared to conventional surfactants which have a smaller number of molecules (monomers) in their aggregates [21]. AMT is used to treat mental or mood difficulties: for example, depression. There are many other applications of this class of drugs, but they also can cause undesired consequences such as anticholinergic, cardiac, and antiarrhythmic side effects. These negative outcomes may be decreased if the drug is fittingly addressed to the organism with the help of a drug carrier. Antidepressant drugs demonstrate association properties and can interact through surfactants, model lipid bilayers, and biomembranes [7]. These drugs can deposit onto biomembranes, which suggests that their pharmacological activities may be associated with membrane interactions. The underlying mechanisms for the numerous biological activities associated with antidepressants might be clarified by analyzing these drug–membrane interactions. Therefore, in the current study, the interactions between the antidepressant drug AMT and the surfactant 14-E2-14 were investigated.

The used AMT drug is soluble in the aqueous solution and formed aggregates structure but at higher concentration. Therefore, needs a carrier for this drug as is not enough hydrophobic. Like other drugs, this drug also showed numerous undesirable’s effects and the use of high concentration of this drug possibly will cause high toxicity means more side effects. Consequently, in the current study ester-bonded gemini surfactant 14-E2-14 is used as a carrier and their mixed micelles with the AMT possibly will enhance their bioavailability and, accordingly, a low concentration of the drug will be needed because *cmc* of the mixed system usually decreases too much (even more than 100 times as compared with pure components).

**Scheme 2:**
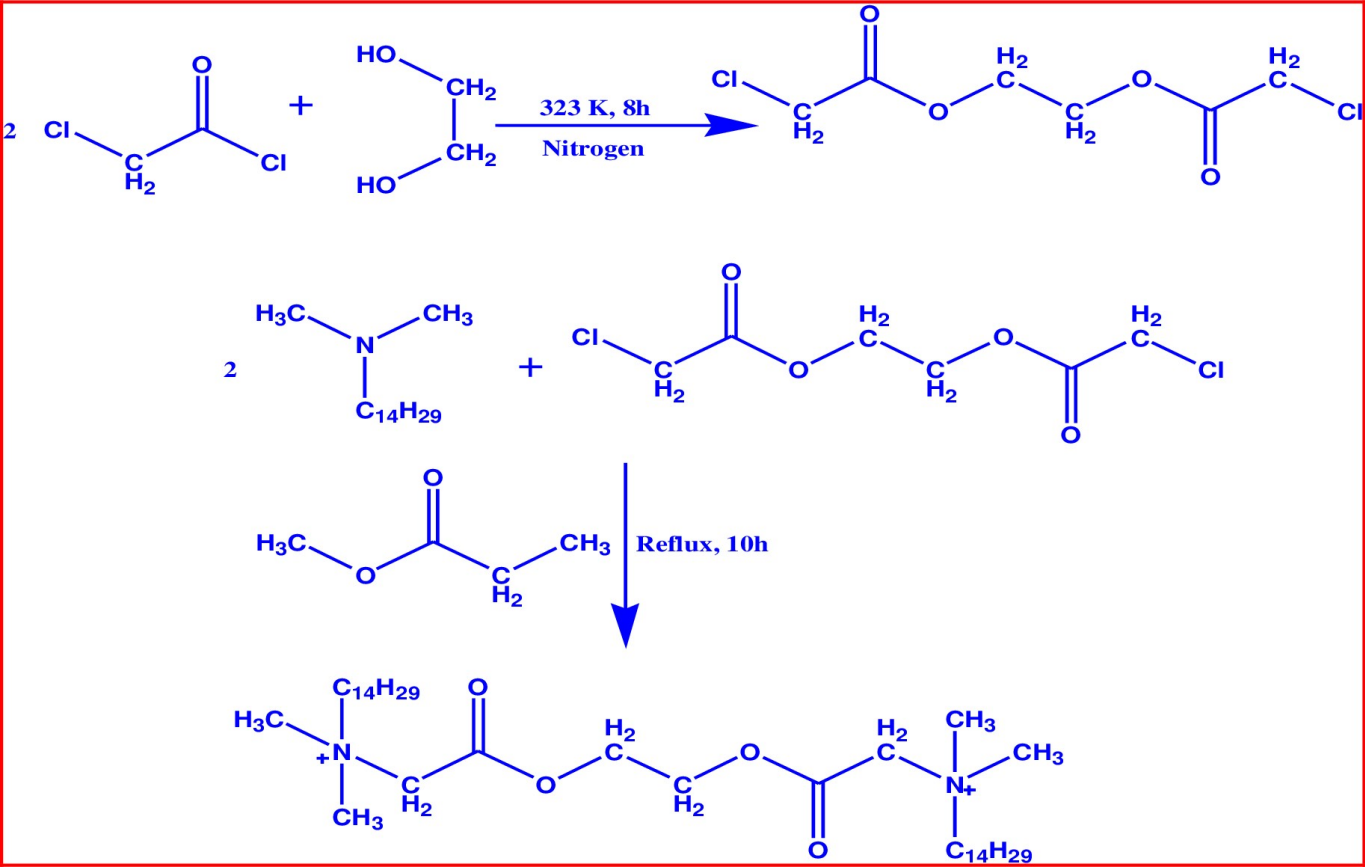
Synthesis pathway of the ester bonded gemini surfactant (14-E2-14).

The literature concerning drug–surfactant interactions concentrates on drugs in combination with conventional ionic and nonionic surfactants (single-chain) [18–20, 22, 23]. Based on previous studies, it is found that the employ of gemini surfactant is showing superior micellar and interfacial properties as compared with conventional surfactants [18–20, 22, 23]. Mahajan et al. [17], studied the interactions of ionic liquids and AMT mixtures in different ratios and evaluated their micellization and interfacial parameters. Alam et al. [18] also evaluated the micellar and interfacial parameters of several drugs in aqueous systems at various concentrations of conventional surfactants using a surface tension measurement. Bagheri and Ahmadi [23] explored the micellization behavior of propranolol hydrochloride drug and conventional surfactant (cetyltrimethylammonium bromide) mixtures in the aqueous system by conductometric methods and evaluated several micellization parameters. In another study, Mahajan et al. [20] has stated the interfacial as well as mixed micellization properties of promethazine hydrochloride drug and a series of pyridinium based gemini surfactant mixtures in an aqueous system using a numerous technique. On comparing their results [18, 19, 22, 23] of the effect of conventional surfactant on the *cmc* of amphiphilic drugs and evaluated different parameters it is found that pyridinium-based gemini surfactant is most effective among all others conventional surfactants [20]. Therefore, here in the current study, a novel biodegradable cationic surfactant, 14-E2-14 was synthesized as mentioned in previous literature (Scheme 2) [14, 16] and examined its interaction with the antidepressant AMT drug in aqueous, NaCl, and urea solutions using a number of techniques. The effect of NaCl and urea is also investigated on the interaction of AMT+14-E2-14 mixtures in this study since salt and urea are found in the human body and may also influence the drug–surfactant interaction and affect the biological activity of the drug. The tensiometry method was employed to evaluate the mixed micellization behavior of AMT+14-E2-14 mixtures. The fluorometry method was utilized to determine the aggregation number (*N*_agg_) and related parameters. Fourier transform infrared (FT-IR) and UV-visible spectroscopy studies were also run to confirm the interaction between the constituents (AMT and 14-E2-14).

## 2. Materials and methods

### 2.1. Materials

All of the materials used are of analytical rating and were used as purchased from their respective supplier without further purification. AMT (≥ 98.0% purity) was purchased from Sigma, USA. NaCl (98.0% purity) was purchased from BDH, England. Urea (98.0% purity) was purchased from Sigma, Germany. Pyrene (99.0% purity) was purchased from Sigma, USA. Cetylpyridinium chloride monohydrate (CPC) was purchased from Merck, Germany. Deionized water with a conductivity of 1.6 x 10^−6^ S cm^-1^ was used for the preparation of stock solutions of AMT and 14-E2-14 in different media solvents (H_2_O/NaCl/urea). Anhydrous salt of CPC was applied subsequently to drying.

### 2.2. Synthesis method of 14-E2-14 surfactants

The cationic gemini surfactant 14-E2-14 was synthesized in the lab as earlier reported as sketched in Scheme 2 which comprised two chief phases [14, 16]. The first phase comprises the preparation of the spacer portion. In the second phase, the 14-E2-14 has attained through heating the products of the first phase i.e., ethane-1,2-diyl bis(chloroacetate) and amine (*N*,*N*-dimethyltetradecylamine) in a molar ratio = 1:2.1) mixture as is reported in the literature [14, 16]. The gemini surfactant obtained in this manner was characterized by numerous analytical techniques. Additionally, precision to the transparency of prepared 14-E2-14 was complete through the tensiometric measurement as no minimum was obtained in surface tension (*γ*) *vs*. log[14-E2-14] plot [2].

### 2.3. Methods

#### 2.3.1. Tensiometric method

The determination of the surface tension (γ) of samples was carried out with an Attension tensiometer (Sigma 701, Germany) using the ring detachment method. The γ values of prepared stock solution were determined through the addition of a fixed amount of AMT, 14-E2-14, or AMT+14-E2-14 mixtures via micropipette in aqueous, NaCl, and urea systems at a temperature of 298.15 K. Measurements were duplicated until the γ was constant. The obtained *γ* of AMT/14-E2-14/AMT+14-E2-14 in aqueous, NaCl, and urea were plotted versus log concentration (log[AMT]/ log[14-E2-14]/ log[AMT+14-E-14]) and a cut-off spot was obtained in the graph and this point is considered as the *cmc* value. The error in temperature and *γ* was found to be ±0.2 K and ±0.2 mNm^-1^ respectively.

#### 2.3.2. FT-IR technique

The FT-IR spectra of the AMT, 14-E2-14, and AMT+14-E2-14 mixed systems in aqueous solution were measured using a Thermo Scientific NICOLET iS50 FT-IR spectrometer (Madison, USA). Spectra were collected between 4000 and 400 cm^–1^ wavelength but only a selected region is shown for clarity. In the case of the stock solutions of mixed systems, only equal ratio mixtures were prepared for the FT-IR measurement. A water background spectrum was subtracted from all spectra collected.

#### 2.3.3. UV-visible study

A Thermo Scientific, Evolution 300 UV-visible spectrometer was employed to record the UV–visible absorbance of AMT solutions by means of increasing the amount of 14-E2-14 in the aqueous system at a temperature of 298.15 K. For baseline correction, deionized water was utilized. The absorbance spectra were noted after every addition of 14-E2-14.

#### 2.3.4. Fluorescence technique

Fluorescence measurements were made to evaluate the aggregation number (*N*_agg_) as well as other related parameters in the absence and presence of fixed concentrations of NaCl and urea using a Hitachi F-7500 fluorescence spectrometer. The fluorescence measurements were recorded by fixing the excitation and emission slit widths at 2.5 nm. A quartz cuvette cell with a 10-mm path length was employed as a sample holder. The spectra were recorded between 350–450 nm by keeping the excitation wavelength fixed at 335 nm. For this study, the concentration of prepared stock solutions was kept just above their respective *cmc* values as obtained from tensiometric measurement and pyrene (PY) solution was utilized as a solvent in place of distilled water. The PY also worked as a probe and CPC was utilized for quenching.

## 3. Results and discussion

### 3.1. Measurement of cmc and cmc^id^ values in different solvent

**Fig 1:**
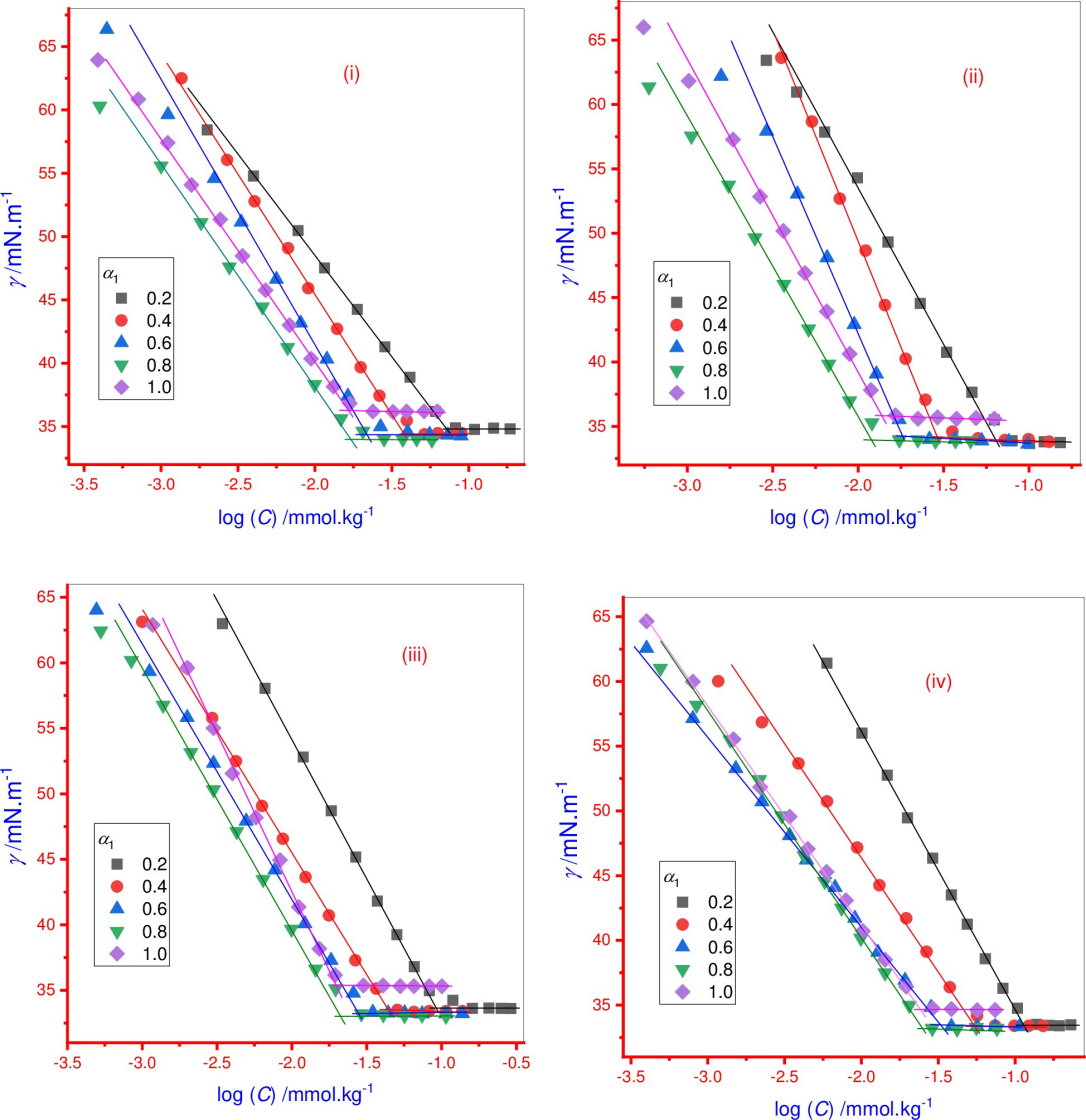
Surface tension (*γ*) versus concentration (*C*) plot for pure 14-E2-14 and AMT+14-E2-14 mixtures in various ratios at 298.15 K: (i) aqueous, (ii) 0.050 mol∙kg^-1^ NaCl, (iii) 0.50 mol∙kg^-1^ urea, and (iv) 1.0 mol∙kg^-1^ urea system.

**Fig 2:**
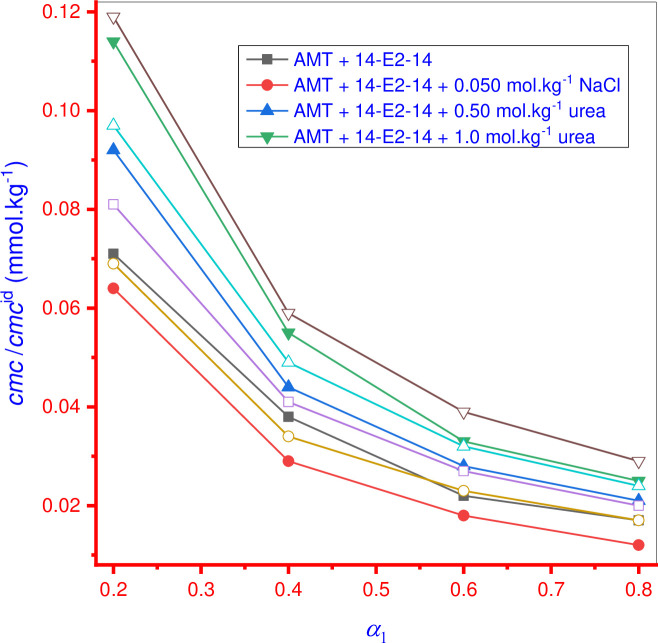
Change of *cmc*/*cmc*^id^ value of AMT+14-E2-14 mixed system vs. mole fraction (*α*_1_) of 14-E2-14 (filled symbol for experimental *cmc* and open symbol for *cmc*^id^).

The tensiometric technique was employed for surface tension (*γ*) measurements of AMT, 14-E2-14 and AMT+14-E2-14 mixture solutions in four different mole fractions (*α*_1_) of 14-E2-14 (0.2 14-E2-14: 0.8 AMT; 0.4 14-E2-14: 0.6 AMT; 0.6 14-E2-14: 0.4 AMT; and 0.8 14-E2-14: 0.2 AMT) to evaluate the *cmc* in aqueous/0.050 mol·kg^-1^ NaCl/0.50 and 1.0 mol·kg^-1^ urea at 298.15 K. The value of the surface tension (*γ*) is linearly associated to the amphiphile concentration in pre-micellar solutions. The value of *γ* continuously decreases with increasing amphiphile concentration and after the micellar regime is reached, *γ* is constant and remains constant with a further addition of amphiphile. The amphiphile concentration corresponding to this break point denotes the *cmc* of amphiphiles (Fig 1). Fig 2 shows the *cmc* of individual 14-E2-14 and AMT+14-E2-14 mixed system in aqueous, NaCl, and urea systems and their obtained values are given in Table 1. The current study is focused on the physicochemical interactions between AMT and potential surfactant carriers, such as 14-E-14, using various theoretical models.

**Table 1:** Various physicochemical parameters for AMT+14-E2-14 mixed system in different solvent at 298.15 K.

*α*_1_	*cmc* (mmol·kg^-1^)	*cmc*^id^ (mmol·kg^-1^)	$X_{1}^{m}$	$X_{1}^{\mathrm{id}}$	*β*^m^	$f_{1}^{m}$	$f_{2}^{m}$	ln(*cmc*_1_/*cmc*_2_)
Aqueous solution								
0	32.36							
0.2	0.072	0.081	0.9077	0.9980	-4.82	0.9598	0.0188	
0.4	0.038	0.041	0.9459	0.9992	-4.87	0.9858	0.0128	-7.59
0.6	0.022	0.027	0.8892	0.9997	-7.62	0.9107	0.0024	
0.8	0.017	0.020	0.9031	0.9999	-8.43	0.9239	0.0010	
1	1.63x10^-2^							
0.050 mol∙kg^-1^ NaCl								
0	29.75							
0.2	0.064	0.069	0.9407	0.9981	-4.01	0.9860	0.0288	
0.4	0.029	0.034	0.8982	0.9993	-6.40	0.9358	0.0057	-7.68
0.6	0.018	0.023	0.8795	0.9997	-8.04	0.8898	0.0020	
0.8	0.012	0.017	0.8571	0.9999	-10.20	0.8116	0.0005	
1	1.38x10^-2^							
0.50 mol∙kg^-1^ urea								
0	36.31							
0.2	0.092	0.097	0.9515	0.9979	-3.55	0.9917	0.0402	
0.4	0.044	0.049	0.9280	0.9992	-5.36	0.9726	0.0099	-7.53
0.6	0.028	0.032	0.9104	0.9996	-6.87	0.9463	0.0034	
0.8	0.021	0.024	0.9138	0.9999	-7.93	0.9428	0.0013	
1	1.95x10^-2^							
1.0 mol∙kg^-1^ urea								
0	39.80							
0.2	0.114	0.119	0.9591	0.9976	-3.20	0.9947	0.0525	
0.4	0.055	0.059	0.9381	0.9991	-4.94	0.9812	0.0129	-7.42
0.6	0.033	0.039	0.8958	0.9996	-7.20	0.9248	0.0031	
0.8	0.025	0.029	0.9033	0.9998	-8.18	0.9264	0.0013	
1	2.39x10^-2^							

At room temperature (298.15 K), the *cmc* of AMT is found to be 32.36 mmol∙kg^-1^, in agreement with the previously reported value in aqueous solution (Table 1) [7, 17, 24]. The value of *cmc* of 14-E2-14 in an aqueous system measured was 1.63x10^-2^ mmol∙kg^-1^, which also achieved an agreement with the literature (Table 1) [14]. The *cmc* value of 14-E2-14 is much less than the *cmc* value of AMT. This occurs because the hydrophobic part of 14-E2-14 is much larger than the hydrophobic part of AMT, and therefore, 14-E2-14 forms micelles at much lower concentration (see Schemes 1, 2). The presence of two head groups of alike charge (catatonic) joined through an ester linkage spacer is another reason for the much lower *cmc* of 14-E2-14, caused by the hindering of the electrostatic repulsion amongst headgroups. Similarly, the O-atoms of the ester linkage spacer form an H-bond to the H_2_O molecules, which decreases the adverse interaction of the hydrophobic chain of the gemini surfactant with the surrounding water. This hydration in the locality of the spacer lessens the electrostatic repulsion between the hydrophilic head groups [25].

The *cmc* values of pure AMT, pure 14-E2-14, and AMT+14-E2-E2 mixed systems of varying *α*_1_ of 14-E2-14 were found to be reduced in NaCl solution relative to in the aqueous system. As it is known that electrolytes encourage micelle formation through lessening the electrostatic interaction amongst the head groups and thus diminishing the effective area occupied by each head group [2]. This reduction of the repulsive interactions amongst head groups of amphiphilic monomers eases micelle formation. The addition of NaCl results in lower *cmc* values of the studied systems because a reduction of electrostatic interactions occurs and hence enhances interactions among the monomers, causing the association to start at lower concentration [26]. Therefore, I can say that more micelles were formed in salt solutions, along with which a rise in the aggregation number (*N*_agg_) occurred [26].

However, in the presence of urea at concentrations of 0.50 and 1.00 mol·kg^-1^ the *cmc* value of systems (AMT, 14-E2-14, and AMT+14-E2-14 mixture) was increased and the values obtained are given in Table 1. As in the previous literature, two dissimilar mechanisms describe the effect of urea on the aqueous system of amphiphiles [27, 28]. The first is the indirect mechanism in which the urea breaks the water structure and the second is the direct mechanism in which urea contributes to the solvation of the hydrophobic chains in the aqueous system by substituting for a number of water particles in the solute hydration shell. Conventionally, the effect of urea on the micellization behavior of amphiphiles is to weaken hydrophobic interactions, meaning that urea acts as a water structure breaker [29]. Urea keeps the capability to stabilize the amphiphile molecules, while also improving the solubility of hydrocarbons in an aqueous system. Furthermore, repulsive interactions among the polar head group molecules at the micellar surface increased in urea solution. Consequently, the start of aggregation of AMT, 14-E2-14, and AMT+14-E2-14 mixed system is delayed when compared with the aqueous solution, and with increasing the concentration of urea (0.50 mol·kg^-1^ to 1.0 mol·kg^-1^), the *cmc* value of systems were further increased. The increase in *cmc* of all system with urea concentration occurred due to the enrichment of the solubility of the nonpolar tail and the increase of solvation of hydrophilic moieties in the presence of urea, owing to association amongst urea and hydrophilic moieties.

Table 1 shows that as the molar fraction (*α*_1_) of 14-E2-14 increased in the AMT+14-E2-14 mixtures, the *cmc* values decreased in all employed media (aqueous/NaCl/urea), indicating that the observed decrease in the *cmc* values of theses mixtures was likely due to the increased interactions between the constituents (AMT and 14-E2-14). As can also be seen from Table 1, *cmc* values of AMT+14-E2-14 mixtures in all solvents were near the *cmc* value of singular 14-E2-14. This phenomenon signifies that the component having higher hydrophobicity starts micelle formation at a lower concentration. In this study, 14-E2-14 was found to be more hydrophobic than AMT, indicating that 14-E2-14 forms micelles at lower concentration and AMT only penetrates into micelles formed by 14-E2-14, suggesting that AMT only supported the formation of mixed micelles. Therefore, it is concluded that mixed micelles of AMT+14-E2-14 mixture are a rich source of 14-E2-14 constituents.

For binary mixed system, the ideal *cmc* values (*cmc*^id^) of the mixed micelles were evaluated using Clint’s model [30], which defines *cmc*^id^ and experimental *cmc* values of singular constituents (*cmc*_1_ and *cmc*_2_) as follows using Eq (1).

$$\frac{1}{cmc^{\mathrm{id}}}=\frac{\alpha_{1}}{cmc_{1}}+\frac{\alpha_{2}}{cmc_{2}}$$ (1)

where *α*_1_ and *cmc*_1_ denotes the mole fraction and *cmc* of 14-E2-14 and *α*_2_ and *cmc*_2_ is the same for AMT. All the *cmc*^id^ calculated for the entire systems are given in Table 1. The deviation of the experimentally obtained *cmc* value from the theoretically obtained *cmc*^id^ accounts for the attractive or repulsive interactions between AMT and 14-E2-14. If a positive deviation i.e., *cmc* > *cmc*^id^, is obtained, then the system will show a negative interaction (repulsion or antagonism) between the components. However, if for any system *cmc* < *cmc*^id^, then the system shows a negative deviation means system showing synergistic or attractive interactions. Finally, if *cmc* = *cmc*^id^, the components of the mixture neither interact nor repel each other.

In the cases studied in this work, the experimental *cmc* of AMT+14-E2-14 mixtures at several *α*_1_ of 14-E2-14 were found to be less than the corresponding value of *cmc*^id^, showing that attractive interaction or synergistic behavior existed among the components (Table 1) as well as systems show nonideal behavior in all studied solvent. On the other hand, the outcomes of these experiments signify that mixed micelles were formed at lower concentrations than expected from their ideal behavior, which suggests good interaction between AMT and 14-E2-14. During the interaction between AMT and 14-E2-14, electrostatic interactions amid the head groups in the micelle, and chain-chain interactions amid dissimilar chain lengths of micelle components took place [31]. Overall, in the NaCl solutions, the deviation of the *cmc* value of the AMT+14-E2-14 mixture from the calculated *cmc*^id^ was greater than in the aqueous system in nearly all, indicating that the salt system shows more nonideal behavior, whereas the *cmc* deviation from *cmc*^id^ in the U system decreases further with an increase in urea concentration. Non-ideality in the studied system is found in following order: AMT+14-E2-14+NaCl > AMT+14-E2-14+H_2_O > AMT+14-E2-14+0.5 mol·kg^-1^ urea > AMT+14-E2-14+1.0 mol·kg^-1^ urea.

### 3.2. Mixed micellization parameter of AMT and 14-E2-14 mixtures

The interactions amongst employed drug and surfactant in all solvents occurred either at the interfacial surface or in the inner side of aggregated structures i.e., micelles. The absorption of the drug in presence of surfactant could be enhanced were understood via consideration of the physicochemical interactions amongst drugs and surfactants. Thus, herein, a systematic study was performed to define the interactions between AMT and 14-E2-14 in various media. The interaction between amphiphiles in mixed micelles was first described by Rubingh’s model [32] which is based on regular solution theory. This model is broadly used due to its simplicity as well as accuracy and is as a basis for the examination of attractive interaction or synergistic outcomes of amphiphiles of mixed systems. In the case of non-ideal solution binary mixtures, the micellar mole fractions of the first component i.e., 14-E2-14 ($X_{1}^{m}$) can be determined through solving Eq (2) [32, 33]:

$$\frac{{\left(X_{1}^{m}\right)}^{2}\ln[\left(\alpha_{1}cmc/X_{1}^{m}cmc_{1}\right)]}{\left(1-X_{1}^{m}{)}^{2}\ln[(1-\alpha_{1})cmc/(1-X_{1}^{m})cmc_{2}\right]}=1$$ (2)

In the ideal solution mixture, the micellar mole fraction of 14-E2-14 in the ideal state ($X_{1}^{\mathrm{id}}$) was calculated using Eq (3), proposed by Motomura [34].

$$X_{1}^{\mathrm{id}}=\frac{\alpha_{1}cmc_{2}}{\alpha_{1}cmc_{2}+\alpha_{2}cmc_{1}}$$ (3)

The values of $X_{1}^{m}$ and $X_{1}^{\mathrm{id}}$ were calculated to compare real and ideal models and their obtained values are listed in Table 1. As shown in Table 1, the calculated values of $X_{1}^{m}$ were found to be less than the values calculated from $X_{1}^{\mathrm{id}}$ for all *α*_1_ in aqueous, NaCl, and urea media indicating that the concentration of 14-E2-14 in mixed micelles was less than expected from their ideal behavior. This means that a number of AMT monomers participate in mixed micelle formation but much fewer than compared with 14-E2-14 [35]. Additionally, the difference between $X_{1}^{m}$ and $X_{1}^{\mathrm{id}}$ values confirm the variance of AMT+14-E2-14 mixtures from ideal behavior, and the results obtained show that 14-E2-14 monomers play a large role in mixed micelle formation. The obtained $X_{1}^{m}$ value was greater in every system than the added *α*_1_ of 14-E2-14, this fact also suggests that the AMT+14-E2-14 mixed micelles contain a higher content of 14-E2-14 (more than 85%) and rest is the drug ($X_{2}^{m}=(1-X_{1}^{m})$) (Table 1). The computed values of $X_{1}^{\mathrm{id}}$ were always found to be more than the *α*_1_ values showing that their value off-course will be more than the $X_{1}^{m}$. Also, $X_{1}^{\mathrm{id}}$ increases with an increase in *α*_1_ of 14-E2-14. In this system, the values of $X_{1}^{m}$ in NaCl or urea solvents do not show any trends with increasing *α*_1_ (Table 1).

The $X_{1}^{m}$ values obtained through the use of Eq (2) were further utilized to evaluate the extent of the interaction between constituents of the mixture, using an interaction parameter, *β*^m^. The *β*^m^ values were evaluated using following Eq (4) [32, 36].

$$\beta^{m}=\frac{\ln(cmc\alpha_{1}/cmc_{1}X_{1}^{m})}{(1-X_{1}^{m}{)}^{2}}$$ (4)

The *β*^m^ value indicates the degree of interaction between amphiphiles as well as the deviation of the real system mixed micelle formation from the ideal behavior. All *β*^m^ values in aqueous along with those in NaCl and urea media are tabulated in Table 1. A positive *β*^m^ value in any binary mixture system shows antagonistic behavior during the formation of mixed micelles. When *β*^m^ = 0 in a binary mixture system, then it is concluded that the system displays ideal behavior during mixed micelle formation. However, a negative *β*^m^ value in any system corresponds to an attractive interaction or synergistic effect amongst the amphiphiles. As given in Table 1, *β*^m^ values were negative in all studied media. Negative *β*^m^ values increase regularly with increasing *α*_1_ of 14-E2-14, showing that only attractive interactions were found, and they increase with increasing *α*_1_ of 14-E2-14 in all cases. As stated above, after mixing the constituents, the obtained negative *β*^m^ values confirmed the existence of attractive interactions. However, before mixing, the repulsive interactions between the constituents were stronger, whereas after mixing, the attractive interactions became dominant over repulsive interaction. The range of *β*^m^ value was found from –10 to –4 in all cases, viewing the high attractive interactions or synergistic effect amongst the constituents. A negative value of *β*^m^ in AMT+14-E2-14 mixed system is due to the hydrophobic attractive interactions amid the hydrophobic portions of the constituents, that leads to a surge of a hydrophobicity along with a lessening of hydrophilicity. The penetration of AMT molecules within the 14-E2-14 micelles reduces the repulsive interaction amongst head groups, moreover, the surging of electrostatic stabilization takes place [37].

Negative values of *β*^m^ indicates attractive interactions. However, synergistic effects in a binary mixed system have been found if the system follows the following two conditions, otherwise, the system shows attractive interaction. The first condition is that the *β*^m^ value should be less than zero and the second condition is that |*β*^m^| is higher than |*ln*(*cmc*_1_/*cmc*_2_)| in all cases. Here only the first rule is fulfilled at all selected *α*_1_ of 14-E2-14. The second condition is only met at *α*_1_ = 0.8 of 14-E2-14. Therefore, attractive interactions can be assumed among the components for the first three molar fractions of 14-E2-14 (*α*_1_), whereas, for the highest molar fraction of 14-E2-14 (*α*_1_), the interactions are likely due to synergistic effects.

In salt solutions, the *β*^m^ values were greater at all *α*_1_ of 14-E2-14, indicating that the attractive interactions between AMT and 14-E2-14 increase with an increase in *α*_1_ of 14-E2-14 in comparison to the aqueous system because the presence of salt drives a decline in repulsive interactions amongst components. Consequently, the negative values of *β*^m^ are enhanced, and also a decrease in the *cmc* of mixed systems occurs with an increase in *α*_1_ of 14-E2-14 in salt system (Table 1). However, in the 0.50 mol·kg^-1^ urea solution, the negative values of *β*^m^ were lower than in the aqueous solution, signifying that in the presence of urea, the interaction amongst constituents reduces (Table 1). This lessening in negative *β*^m^ values occurred because urea molecules join spontaneously through the hydrophobic part of AMT and 14-E2-14 monomers, which lessens the hydrophobicity of the system. This phenomena in the urea solution enhance the *cmc* value accompanied by a decrease in negative *β*^m^ values. With an increase in the concentration of urea (0.5 mol·kg^-1^ to 1.0 mol·kg^-1^), the negative *β*^m^ values were further decreased. Despite this effect, as shown in Table 1, the obtained *β*^m^ values for the AMT+14-E2-14 mixtures in each media were not constant through the variations of *α*_1_ (14-E2-14). This observed non-constancy of *β*^m^ values across various mixture compositions are considered to be a limitation of the Rubingh′s model for mixed binary systems [1].

By knowing the $X_{1}^{m}$ and *β*^m^ values, it is possible to assess the activity coefficients $f_{1}^{m}$ (14-E2-14) and $f_{2}^{m}$ (AMT) through the following equations.

$$f_{1}^{m}=\exp[\beta^{m}(1-X_{1}^{m}{)}^{2}]$$ (5)

$$f_{2}^{m}=\exp\{\beta^{m}(X_{1}^{m}{)}^{2}\}$$ (6)

The calculated values of $f_{1}^{m}$ (14-E2-14) and $f_{2}^{m}$ (AMT)) in the present work are given in Table 1. These values were under 1 in every case, signifying the non-ideal behavior of the mixed system along with attractive interactions between the AMT and 14-E2-14. Furthermore, the $f_{1}^{m}$ of 14-E2-14 is found to be much larger than the $f_{2}^{m}$ of AMT, again confirming that mixed micelles encourage more participation of 14-E2-14 [36]. Herein, the $f_{2}^{m}$ (AMT)) value was decreased with an increase in the *α*_1_ of 14-E2-14. This behavior reveals that the involvement of AMT in mixed micelles decreases with an increase in *α*_1_ of 14-E2-14, showing the difference in the distribution of the constituents (14-E2-14 and AMT) amongst the mixed micelles.

### 3.3. Properties at the air-solution interface of pure and mixed systems

The number of monomers adsorbed at the air-solution interface can be computed via a surface parameter known as the maximum surface excess concentration (*Γ*_max_). *Γ*_max_ is defined as the area of the interfacial surface covered by similar or identical amphiphiles, thereby decreasing the γ of solvent at the *cmc*. For the dilute solution, the values of *Γ*_max_ in aqueous and non-aqueous systems can be assessed through the Gibbs adsorption isotherm [38, 39].

$$\Gamma_{max}=-\frac{1}{2.303nRT}\left(\frac{\partial \gamma }{\partial \log\left(C\right)}\right)\;(mol\;m^{‐2})$$ (7)

In Eq (7), the γ = surface tension in mN·m^−1^, *T* = temperature (K), *R* = universal gas constant, *C* = total concentration of participating amphiphiles in a pure and mixed state, and *n* = total number of species per amphiphile monomer participating during adsorption phenomena [2]. The value of *n* = 2 for singular AMT and in the case of 14-E2-14, *n* = 3. But, for mixed systems, a value of *n* was estimated through the equation: $n=n_{1}X_{1}^{\sigma }+n_{2}(1-X_{1}^{\sigma })$ [40]. $X_{1}^{\sigma }$ is the molar composition in the mixed interface and their value is given in Table 2. The value of slope $\left(\frac{\partial \gamma }{\partial \log\left(C\right)}\right)$ in all cases was evaluated at the chosen concentration to assess the final *Γ*_max_ value.

**Table 2:** Various interfacial parameters for AMT+14-E2-14 mixed system in diverse solvent at 298.15 K.

*α*_1_	*X*_1_^σ^	*β*^σ^	*f*_1_^σ^	*f*_2_^σ^	*Γ*_max_ 10^7^ (mol m^-2^)	*A*_min._/*A*^id^ (Ǻ^2^)	*γ*_cmc_	*π*_*cmc*_ (mN m^-1^)	*pC*_20_	ln(*C*_1_/*C*_2_)
Aqueous solution										
0					20.13	82.49	42.48	28.52	1.87	
0.2	0.8130	-9.22	0.7244	0.00226	9.90	167.71/143.77	34.90	36.10	5.15	
0.4	0.9061	-7.33	0.9374	0.00243	11.50	144.37/150.79	34.52	36.48	5.29	
0.6	0.9031	-8.47	0.9235	0.00099	11.92	139.25/150.57	34.39	36.61	5.47	-8.62
0.8	0.8398	-12.30	0.7294	0.00017	10.62	156.41/145.79	34.15	36.85	5.73	
1					10.52	157.87	36.11	34.89	5.61	
0.050 mol∙kg^-1^ NaCl										
0					20.37	81.49	43.04	27.96	1.86	
0.2	0.8713	-6.83	0.8930	0.00559	13.71	121.13/115.82	33.72	37.28	4.90	
0.4	0.9306	-6.26	0.9703	0.00443	19.15	86.705/118.16	33.99	37.01	5.05	-8.36
0.6	0.9602	-6.13	0.9903	0.00350	17.67	93.979/119.32	33.91	37.09	5.29	
0.8	0.8405	-11.93	0.7383	0.00022	14.18	117.10/114.61	33.89	37.11	5.65	
1					13.73	120.89	35.85	35.15	5.49	
0.50 mol∙kg^-1^ urea										
0					18.57	89.41	42.43	28.57	1.84	
0.2	0.8271	-7.89	0.7897	0.00451	13.33	124.55/116.87	33.57	37.43	4.84	
0.4	0.7819	-11.48	0.5793	0.00090	11.93	139.15/115.37	33.32	37.68	5.30	-8.09
0.6	0.7933	-12.25	0.5925	0.00045	12.12	136.97/115.75	33.19	37.81	5.46	
0.8	0.8077	-13.09	0.6164	0.00020	12.56	132.20/116.23	33.12	37.88	5.56	
1					13.54	122.61	35.37	35.63	5.35	
1.0 mol∙kg^-1^ urea										
0					14.06	118.05	44.09	26.91	1.83	
0.2	0.8001	-9.74	0.6777	0.00196	13.84	119.98/154.13	33.43	37.57	4.70	
0.4	0.9004	-7.52	0.9282	0.00226	10.67	155.68/158.66	33.35	37.65	5.25	-8.61
0.6	0.7990	-12.79	0.5965	0.00028	9.43	176.19/154.08	33.27	37.73	5.67	
0.8	0.8720	-10.88	0.8367	0.00026	10.65	155.92/157.38	33.20	37.80	5.61	
1					10.18	163.15	34.67	36.33	5.57	

When a monolayer becomes saturated with the addition of amphiphiles then further addition of the compound causes the formation of micelles. The minimum area per monomer (*A*_min_) at the saturated monolayer can be attained from the equation [39, 40]:

$$A_{\min}=\frac{10^{20}}{N_{A}\Gamma_{\max}}({Å}^{2})$$ (8)

In Eq (8), *N*_A_ = Avogadro’s number and the units are Å^2^. The calculated *Γ*_max_ and *A*_min_ values of pure AMT, 14-E2-14, and AMT+14-E2-14 mixed systems in H_2_O, NaCl, and urea are given in Table 2. The obtained *Γ*_max_ and *A*_min_ values of singular 14-E2-14 are found to be 10.52 mol·m^-2^ and 157.87 Ǻ^2,^ respectively, showing somewhat good agreement with the previously stated value [41]. The value of *Γ*_max_ of pure AMT is greater than the *Γ*_max_ value of singular 14-E2-14, which means *A*_min_ shows the reverse trend because these parameters are inversely proportional to each other. A lower value of *Γ*_max_ (or a higher value of *A*_min_) for pure 14-E2-14 was achieved because of the repulsion between the similarly charged head groups present in a single monomer of 14-E2-14 causes distance between them. Accordingly, the spacer residue is fully stretched and hence this occupies more space. In all solvent systems, the *Γ*_max_ value for AMT+14-E2-14 mixtures was found below the *Γ*_max_ value of pure AMT but found to be higher than *Γ*_max_ value of 14-E2-14 with few exceptions. However, *Γ*_max_ did not change consistently in response to increases in *α*_1_ of 14-E2-14.

In the NaCl system, *Γ*_max_ of pure AMT and 14-E2-14 along with AMT+14-E2-14 mixed systems were found to be greater than in the aqueous system (Table 2). This observed increase in the *Γ*_max_ value in the salt solution can be attributed to the weakening of electrostatic repulsions between the components. Therefore, the efficiency of the employed monomers resides at the interface is increased, which enhances the compactness of the molecules at the monolayer/mixed monolayer. In the urea system, the *Γ*_max_ value for pure AMT is found to be decreased at both concentrations of urea, and the value for pure 14-E2-14 decreases only at the higher concentration of urea (1.0 mol·kg^-1^) but there is no trend for AMT+14-E2-14 mixtures. This decrease in *Γ*_max_ value in the urea system is because of the repulsive interactions, which enlarge the head groups at the interfacial surface.

The occupied minimum area per amphiphilic monomer (*A*^id^) under ideal conditions was evaluated using Eq (9).

$$A^{\mathrm{id}}=X_{1}^{\sigma }A_{1}+(1-X_{1}^{\sigma })A_{2}$$ (9)

Here *A*_1_ = *A*_min_ of pure 14-E2-14 and *A*_2_ = *A*_min_ of pure AMT. The *A*_min_ values of AMT+14-E2-14 mixtures are found to be higher in some *α*_1_ of 14-E2-14 while lower in the rest of cases than the corresponding *A*^id^ values (Table 2). The value of *A*_min_ exceeds the corresponding *A*^id^ value due to the rigid and higher hydrophobic volumes of the components which produce steric interruption in all studied solvents.

In parallel to Rubingh’s theory [32] for mixed micelles as in Eqs (2) and (4), Rosen et al. [38, 42] generated a model to assess the composition of mixed adsorbed interfaces and the interaction parameter (*β*^σ^) at the interfacial surface by following the given equations.

$$\frac{{\left(X_{1}^{\sigma }\right)}^{2}\ln[\left(\alpha_{1}C/X_{1}^{\sigma }C_{1}\right)]}{\left(1-X_{1}^{\sigma }{)}^{2}\ln[(1-\alpha_{1})C/(1-X_{1}^{\sigma })C_{2}\right]}=1$$ (10)

$$\beta^{\sigma }=\frac{\ln(C\alpha_{1}/C_{1}X_{1}^{\sigma })}{(1-X_{1}^{\sigma }{)}^{2}}$$ (11)

where *C*_1_ = concentration of pure 14-E2-14, *C*_2_ = concentration of pure AMT, and *C* = concentration of the mixed AMT+14-E2-14 system at the various *α*_1_ of 14-E2-14 needed to produce an assumed surface tension reduction at the interfacial surface. $X_{1}^{\sigma }$ = composition of 14-E2-14 at the mixed monolayer. The $X_{1}^{\sigma }$ and *β*^*σ*^ calculated for all systems are given in Table 2. The obtained values of $X_{1}^{\sigma }$ were found in between the 0.7819 and 0.9602, showing that the interface contains mostly 14-E2-14. Tables 1 and 2 show that the average value of $X_{1}^{\sigma }$ is also to be comparable to the average $X_{1}^{m}$ value, showing that the concentration of 14-E2-14 is nearly the same in the mixed monolayer and mixed micelles. Through the *α*_1_ of 14-E2-14 changes in the mixtures, the $X_{1}^{\sigma }$ value is not representing any fixed trend regarding an increase or decrease in all solvent. In the NaCl solution, the $X_{1}^{\sigma }$ value is higher than in aqueous as NaCl reduced the repulsive interaction between the AMT and 14-E2-14, hence the concentration of 14-E2-14 increases in the mixed monolayer in the presence of salt (Table 2). However, in the urea system, the $X_{1}^{\sigma }$ value declines with few exceptions, as the repulsive interaction amongst AMT and 14-E2-14 mixture increases.

Comparable to *β*^m^, a negative value of *β*^*σ*^ indicates attractive interactions between both kinds of molecules at a monolayer, a positive value of *β*^*σ*^ indicates repulsion amongst both components, and *β*^*σ*^ = 0 is an ideal mixed monolayer. *β*^*σ*^ values were found to be negative in all cases (Table 2), showing attractive interactions between the AMT and 14-E2-14 monomers at the air-solution surfaces. The average *β*^*σ*^ value of all systems is a bit more than the average *β*^m^ value for all the solvents, indicating the interactions among constituents in the mixed monolayer are achieved more than the interactions among constituents in mixed micelles. In NaCl and urea solutions, the value of *β*^*σ*^ does not show any specific trend, but in all cases they are negative.

Antidepressant drug AMT combined with the gemini surfactant 14-E2-14 shows higher surface activities along with much lower *cmc* value than pure AMT. The presence of synergism in binary mixtures not only depends on the strength of the interaction amongst constituents (indicated by *β*^m^ or *β*^*σ*^) but also on the other related characteristics of the distinct constituents of the binary mixture. The conditions for synergistic behavior in γ reduction efficiency [2] are: (1) the concentration of the components desirable to produce a given reduction efficiency in γ of the solution at the interfacial surface and (2) the system should obey the following two conditions.

$$\left(I\right).\;\beta^{\sigma }<0$$

$$\left(II).\;|\beta^{\sigma }|>|ln\;(C_{1}/C_{2})\right|$$

As stated above, the evaluated value of *β*^*σ*^ was found to be negative in all systems at the interface, however |*β*^*σ*^| is not higher than |ln (*C*_1_/*C*_2_)| value at all *α*_1_ of 14-E2-14 means the second condition is not achieved in all cases, and therefore, the AMT and 14-E2-14 mixtures show synergism in *γ* reduction efficiency at only some *α*_1_ of 14-E2-14.

Paralleling the mixed micelles, the activity coefficients (*f*_1_^σ^ (14-E2-14) and *f*_2_^σ^ (AMT)) of both components was also evaluated for the mixed monolayer via the *β*^*σ*^ and $X_{1}^{\sigma }$ parameters through the following equations [2].

$$f_{1}^{\sigma }=\exp\{\beta^{\sigma }(1-X_{1}^{\sigma }{)}^{2}\}$$ (12)

$$f_{2}^{\sigma }=\exp\{\beta^{\sigma }(X_{1}^{\sigma }{)}^{2}\}$$ (13)

The achieved values of *f*_1_^σ^ (14-E2-14) and *f*_2_^σ^ (AMT) of entire cases are given in Table 2. For all solvents, the values are <1, indicating non-ideal behavior for mixed monolayers along with attractive interactions. The activity coefficient results also show that in the mixed monolayer, the contribution of 14-E-14 is much greater than the contribution of AMT because the *f*_1_^σ^ value is greater than the value of *f*_2_^σ^ in all cases.

The efficiency of surface adsorption of any chosen amphiphile solution can be usefully indicated via a parameter *pC*_20_ computed by Eq (14) below [2, 43].

$$pC_{20}=–log\;C_{20}$$ (14)

where *C*_20_ = concentration of compound desired to decrease the *γ* of the solvent by 20 mN·m^−1^. The computed *pC*_20_ values of pure AMT, 14-E2-14, and AMT+14-E2-14 mixed system are recorded in Table 2. A higher *pC*_20_ value shows that a lower concentration of amphiphile is needed to diminish the *γ* value by 20 mN m^–1^ [2]. The obtained results indicated that the calculated *pC*_20_ value of 14-E2-14 was higher than those for AMT in all solvents. This means that 14-E2-14 has higher interfacial adsorption efficiency than AMT. This phenomenon is already proven by their respective *cmc* values. Table 2 also shows that the addition of 14-E2-14 to a solution of AMT causes a significant rise in *pC*_20_ value from that of pure AMT. By enhancing the *α*_1_ of 14-E2-14 in the solution mixture, the interfacial adsorption efficiency of the mixed system increases considerably; however, the *pC*_20_ value of the AMT+14-E2-14 mixture was found to be close to the *pC*_20_ value of pure 14-E2-14. But at the highest *α*_1_ value (14-E2-14), the *pC*_20_ value of the mixture was greater than the *pC*_20_ value of pure 14-E2-14 (Table 2).

Another parameter called the surface pressure at the *cmc* (*π*_cmc_) was analyzed by means of Eq (15) [2]:

$$\pi_{cmc}=\gamma_{0}-\gamma_{cmc}$$ (15)

where γ_0_ = surface tension of individual solvents (water/NaCl/ urea) and γ_*cmc*_ = surface tension at *cmc* of a pure or mixed system. The parameter π_*cmc*_ specifies the efficiency of the system under consideration to decrease the *γ* of the solvent. The *γ*_cmc_ and π_*cmc*_ values for all systems studied are presented in Table 2. The π_*cmc*_ value of 14-E2-14 is higher than that of AMT in all solvents; however, for the AMT+14-E2-14 mixed system, π_*cmc*_ was found to be higher at all mole fractions than π_*cmc*_ of AMT but less than π_*cmc*_ of 14-E2-14.

### 3.4. Thermodynamic parameters

Thermodynamic parameters such as the Gibbs free energy of micellization ($\Delta G_{mic}^{\circ }$) income the moment as the amphiphilic monomers are converted from a bulk solution to the micellar form can be assessed by considering a charged pseudo-phase separation model using the following equation [44–46]:

$$\Delta G_{mic}^{\circ }==RTlnX_{cmc}$$ (16)

In surface tension measurements it is well-documented that the complete dissociation of components has taken place; therefore, here the degree of dissociation is taken as one. *X*_*cmc*_ = *cmc* in mole fraction, *R* and *T* are their traditional values.

Table 3 depicts the calculated $\Delta G_{mic}^{\circ }$ values for neat AMT, neat 14-E2-14, and AMT+14-E2-14 mixed systems in all solvents. All $\Delta G_{mic}^{\circ }$ values were negative, indicating that the formed stable micelles are thermodynamically spontaneous in nature. The negative values of $\Delta G_{mic}^{\circ }$ of AMT+14-E2-14 mixed systems increase with increasing the 14-E2-14 mole fraction and are maximal at the highest *α*_1_ of 14-E2-14 (Table 3), showing that the spontaneity of systems increases with an increase in *α*_1_ of 14-E2-14 also [47]. In pure AMT, $\Delta G_{mic}^{\circ }$ is in good agreement with the published value and the $\Delta G_{m}^{\circ }$ of pure 14-E2-14 is found to be in the same range as earlier stated values [48, 49]. As shown in Table 3, $\Delta G_{mic}^{\circ }$ of 14-E2-14 was found to be much higher than the $\Delta G_{mic}^{\circ }$ of AMT. This is because 14-E2-14 contains a long hydrophobic portion compared with AMT, so in the case of 14-E2-14, the process of micelle formation is more spontaneous. In the NaCl system, the $\Delta G_{mic}^{\circ }$ value in all studied solutions became more negative than in aqueous, demonstrating that the hydrophobicity in the salt system increased because the interactions between like and unlike monomers increase along with a decrease in the number of electrostatic repulsions occurring. Therefore, the micellization process starts at a lower concentration. On the other hand, in the urea system, the $\Delta G_{mic}^{\circ }$ value of all studied solutions became less negative, showing that the interactions between like and unlike monomers decreased. However, association in the pure and mixed compounds is spontaneous in urea, but the magnitude decreases than the aqueous and NaCl systems (Table 3). As the concentration of urea increases from 0.5 to 1.0 mol·kg^-1^, the negative $\Delta G_{mic}^{\circ }$ value of pure and mixed systems further decreased. From the overall data, it is concluded that negative values of $\Delta G_{mic}^{\circ }$ follow an inverse tendency with *cmc* values. For 1.0 mol·kg^-1^ urea, increases the *cmc* of AMT, 14-E2-14, and AMT+14-E2-14 mixed systems more as compared to 0.5 mol·kg^-1^ urea, hence the negative $\Delta G_{mic}^{\circ }$ value of AMT, 14-E2-14, and AMT+14-E2-14 mixed systems in the presence of 0.5 mol·kg^-1^ urea is higher. The addition of urea changes the bulk phase properties making it more favorable than the aqueous system for amphiphilic monomers [50], therefore, the movement of the hydrophobic chain from the bulk phase toward the micellar region is become less favorable, and hence the value of $\Delta G_{mic}^{\circ }$ becomes less negative.

**Table 3:** Different thermodynamic parameters and packing parameter (*P*) for AMT-14-E2-14 mixed system in diverse solvent at 298.15 K.

*α*_1_	$\Delta G_{mic}^{\circ }$ (kJ mol^-1^)	$\Delta G_{\mathrm{ad}}^{\circ }$ (kJ mol^-1^)	*G*_min_ (kJ mol^-1^)	$\Delta G_{\mathrm{ex}}^{m}$ (kJ mol^-1^)	$\Delta G_{\mathrm{ex}}^{\sigma }$ (kJ mol^-1^)	*P*
Aqueous system						
0	-18.45	-32.62	21.10			0.54
0.2	-33.62	-70.08	35.25	-1.01	-3.47	0.26
0.4	-35.17	-66.89	30.02	-0.62	-1.55	0.30
0.6	-36.52	-67.23	28.84	-1.86	-1.84	0.31
0.8	-37.16	-71.88	32.17	-1.83	-4.10	0.28
1	-37.26	-70.44	34.33			0.28
0.050 mol∙kg^-1^ NaCl						
0	-18.66	-32.38	21.13			0.54
0.2	-33.88	-61.07	24.60	-0.55	-1.90	0.36
0.4	-35.84	-55.16	17.75	-1.45	-1.01	0.50
0.6	-37.02	-58.01	19.19	-2.11	-0.58	0.46
0.8	-38.02	-64.2	23.90	-3.10	-3.96	0.37
1	-37.68	-63.27	26.10			0.36
0.50 mol∙kg^-1^ urea						
0	-18.17	-33.55	22.85			0.50
0.2	-32.98	-61.05	25.18	-0.41	-2.80	0.35
0.4	-34.80	-66.38	27.92	-0.89	-4.85	0.31
0.6	-35.92	-67.12	27.38	-1.39	-4.98	0.32
0.8	-36.64	-66.8	26.37	-1.55	-5.04	0.33
1	-36.82	-63.13	26.12			0.36
1.0 mol∙kg^-1^ urea						
0	-17.94	-37.07	31.35			0.38
0.2	-32.45	-59.59	24.16	-0.31	-3.86	0.36
0.4	-34.25	-69.55	31.27	-0.71	-1.67	0.28
0.6	-35.52	-75.56	35.31	-1.66	-5.09	0.25
0.8	-36.20	-71.7	31.18	-1.77	-3.01	0.28
1	-36.32	-72.02	34.07			0.27

Furthermore, the obtained $\Delta G_{mic}^{\circ }$ value can be incorporated into the standard Gibbs energy of adsorption ($\Delta G_{\mathrm{ad}}^{\circ }$) at the interfacial surface to evaluate their values for neat and mixed systems by means of the equation given below [51, 52].

$$\Delta G_{\mathrm{ad}}^{\circ }=\Delta G_{mic}^{\circ }-\frac{\pi_{\mathrm{cmc}}}{\Gamma_{max}}$$ (17)

In Eq (17), *π*_cmc_ shows the surface pressure at *cmc*. The term ($\frac{\pi_{\mathrm{cmc}}}{\Gamma_{max}}$) signifies the work at a zero-surface pressure, the work comprised of transporting the amphiphilic monomers from an interfacial monolayer to the micelle. But herein, the obtained value of $\frac{\pi_{\mathrm{cmc}}}{\Gamma_{max}}$ is much smaller than $\Delta G_{mic}^{\circ }$, which means that at zero surface pressure the work required to transport the amphiphile monomers from an interfacial surface to the micelle is insignificant.

Here the obtained values of $\Delta G_{\mathrm{ad}}^{\circ }$ are negative for AMT, 14-E2-14, and AMT+14-E2-14 mixtures in all solvents, showing that component adsorption at the interfacial surface occurs spontaneously (Table 3). Furthermore, the $\Delta G_{\mathrm{ad}}^{\circ }$ values were more negative than $\Delta G_{mic}^{\circ }$ indicating that once a micelle is formed, some extra work is needed to transfer the amphiphile monomers from the air-solvent interface into the micellar form as well as also showing that adsorption is more favorable [53]. The $\Delta G_{\mathrm{ad}}^{\circ }$ value of pure AMT in all studied solvents is found to be less negative than the $\Delta G_{\mathrm{ad}}^{\circ }$ value of pure 14-E2-14 and AMT+14-E2-14 mixtures of all ratios in all solvents; however, the $\Delta G_{\mathrm{ad}}^{\circ }$ value of 14-E2-14 is found to be approximately equal to AMT+14-E2-14 mixtures at lower *α*_1_ of 14-E2-14, but at higher *α*_1_, $\Delta G_{\mathrm{ad}}^{\circ }$ of mixed systems was greater than the $\Delta G_{\mathrm{ad}}^{\circ }$ value of pure14-E2-14 (Table 3). The $\Delta G_{\mathrm{ad}}^{\circ }$ value reveals that the adsorption phenomena in the mixture is easier than adsorption of pure AMT and that the spontaneity of mixed systems increases with a rise in *α*_1_ of 14-E2-14. Moreover, the more negative $\Delta G_{\mathrm{ad}}^{\circ }$ compared with $\Delta G_{mic}^{\circ }$ shows that the adsorption phenomena is favored over the association phenomena in bulk systems because of the hydrophobic part of the components, which preferred monomers to the interfacial surface. In the salt or urea system, the $\Delta G_{\mathrm{ad}}^{\circ }$ value of pure and mixed systems at all ratios does not show any definite trend (Table 3).

Another thermodynamic parameter known as excess free energy (*ΔG*_ex_) values were computed using the following equations [54–57]. This parameter for mixed micellization is symbolized by $\Delta G_{\mathrm{ex}}^{m}$ and for mixed monolayer is designated by $\Delta G_{\mathrm{ex}}^{\sigma }$ and their values were evaluated using the given equations.

$$\Delta G_{\mathrm{ex}}^{m}=RT[X_{1}^{m}\;\ln\;f_{1}^{m}+(1-X_{1}^{m})\;\ln\;f_{2}^{m}]$$ (18)

$$\Delta G_{\mathrm{ex}}^{\sigma }=RT[X_{1}^{\sigma }\;\ln\;f_{1}^{\sigma }+(1-X_{1}^{\sigma })\;\ln\;f_{2}^{\sigma }]$$ (19)

The calculated values of $\Delta G_{\mathrm{ex}}^{m}$ and $\Delta G_{\mathrm{ex}}^{\sigma }$ of AMT+14-E2-14 mixed system in all solvent media (aqueous/NaCl/urea) are shown in Table 3. All values are negative for every case, signifying the higher stabilization of mixed micelle and mixed monolayer formation as compared with micelles and monolayers formed from single components. Overall, but not in all cases, at the highest *α*_1_ of 14-E2-14, $\Delta G_{\mathrm{ex}}^{m}$ and $\Delta G_{\mathrm{ex}}^{\sigma }$ were higher than those of lower *α*_1_ of 14-E2-14, which means that introduction of high concentrations of 14-E2-14 monomers makes the mixed micelles more stable. By comparing the values of $\Delta G_{\mathrm{ex}}^{\sigma }$ and $\Delta G_{\mathrm{ex}}^{m}$, it is seen that the average value of $\Delta G_{\mathrm{ex}}^{\sigma }$ is obtained greater than the average value of $\Delta G_{\mathrm{ex}}^{m}$, signifying that the mixed monolayer shows some extra stability (Table 3). In salt and urea solutions, excess free energy values do not follow a definite trend.

During the formation of the mixed monolayer, the attractive interaction can be computed by means of an alternative thermodynamic measure, called the minimum free energy (*G*_min_), of an interface at maximum adsorption, which is evaluated using the following equation [58].

$$G_{min}=\gamma_{cmc}A_{min}N_{A}$$ (20)

In the above equation *γ*_*cmc*_ is the surface tension of the amphiphile at equilibrium (at *cmc*). *G*_*min*_ is the change in free energy in consort when molecules shift from the bulk system to the interface, or the effort required to form an interface per mole. The lesser *G*_*min*_ value shows the formation of the thermodynamically more stable surface, which is directly proportional to the attractive interaction amongst components. The lower value of *G*_*min*_ calculated for neat AMT, 14-E2-14, as well as AMT+14-E2-14 mixed systems in different ratio in all studied solvents, indicates that thermodynamically stable interfaces are formed (Table 3). This indicates that interactions between AMT and 14-E2-14 are valuable. However, the *G*_min_ value in the system does not behave consistently with variation in *α*_1_, and a similar lack of trend is observed in the NaCl and urea systems (Table 3).

### 3.5. Packing parameters of studied systems

In aqueous as well as non-aqueous systems, the packing parameter regarding the shape of the formed micelles, *P*, is evaluated through Tanford’s formula [59]:

$$P=\frac{V_{0}}{A_{min}l_{c}}$$ (21)

where *V*o is the hydrophobic chain volume in the core of micelles, *lc* is the length of the hydrocarbon chain of the micelles core and *A*_min_ is the minimum area per monomer at the interfacial surface. The values of *V*o and *lc* can be computed by following Tanford’s equations [59]:

$$V_{0}=[27.4+26.9\;(n_{c}-1)]\;x\;2\;({Å}^{3})$$ (22)

$$l_{c}=[1.54+1.26\;(n_{c}-1)]\;(Å)$$ (23)

where *n*_c_ is the total number of C-atoms in the hydrocarbon chain. During the calculation of *V*o and *lc* values, the overall count of carbon atoms in the hydrocarbon chain is taken to be one fewer than the real number of carbon atoms because the carbon attached to the head group is exceedingly solvated, therefore this carbon atom is also treated as a part of the head group [60]. Evaluated *P* values of all systems (AMT, 14-E2-14, and AMT+14-E2-14 mixtures) in aqueous and the presence of NaCl and urea are given in Table 3.

Furthermore, it is reported that for *P* = 0 to 0.33 the shape of the formed aggregate is spherical. For *P* = 0.33 to 0.5 the micellar shape is cylindrical. But for *P* = 0.5 to 1, the formed micellar shape is vesicular [2]. Table 3 shows that for case singular AMT, *P* value was obtained above 0.50 with the exception in 1.0 mol·kg^-1^ urea system, recommends that the AMT forms vesicles shape micelles, and in 1.0 mol·kg^-1^ urea system AMT form cylindrical shape micelles. In the case of AMT+14-E2-14 mixture in different ratio was found amidst the 0.25 to 0.50, viewing that in some case micelles formed are spherical shape and in some other case the formed micelles are cylindrical (mainly in aqueous and in 1.0 mol·kg^-1^ urea system, formed mixed micelles are spherical). For pure 14-E2-14, the formed micelles are spherical in aqueous/1.0 mol·kg^-1^ urea system and found to be cylindrical in shape in NaCl and 0.50 mol·kg^-1^ urea system.

### 3.6. FT-IR study

**Fig 3:**
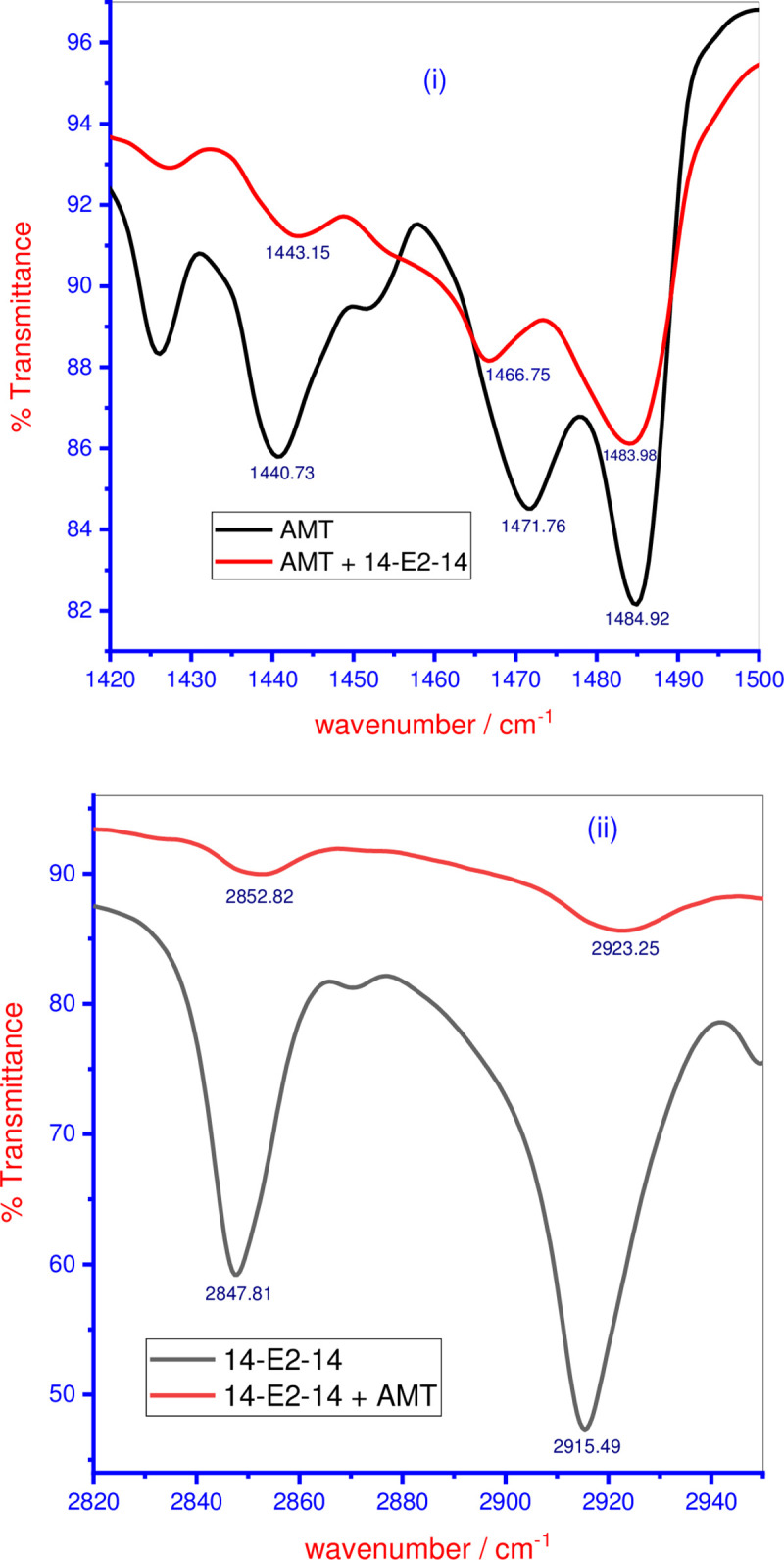
FTIR spectra of (i) AMT in absence and presence of 14-E2-14 and (ii) 14-E2-14 in absence and presence of AMT (both mixtures are in equal ratio).

The FT-IR technique is another good method to evaluate the interaction within the binary mixed system which forms mixed micelles [61]. This analysis was taken on to investigate and determine the structural information of the prevailing intermolecular interactions. The FT-IR spectra of AMT and AMT+14-E2-14 mixed system in an equal ratio in the aqueous system is presented in Fig 3(i) for the region of 1490 cm^-1^ to 1420 cm^-1^. The pure AMT molecule is comprised of a positively charged N-atom to which two methyl (-CH_3_) groups along with one methylene (-CH_2_-) is connected. The conceivable interactions between AMT and 14-E2-14 occur due to the shifts in the -C-H bending frequency of the methyl groups as well as the methylene group in the head group region of AMT. For pure AMT, the FT-IR bands are observable at 1484.92, 1471.76, and 1440.73 cm^-1^, allocated to C-H bending of methyl and the methylene group. The -CH bending bands of methyl and methylene of AMT in the presence of 14-E2-14 were shifted from their original position in the following manner: 1484.92 to 1483.98 cm^-1^, 1471.76 to 1466.74 cm^-1^_,_ and 1440.73 to 1443.15 cm^-1^. These frequency shifts suggest a slightly higher ordering of the hydrophobic part of the AMT molecules in the mixed micelles formed by AMT+14-E2-14 because of the strong interaction of AMT with 14-E2-14 (Fig 3(i)) [57, 62].

The FT-IR spectra of pure 14-E2-14, as well as 14-E2-14+AMT mixed system in equal ration of aqueous system, is depicted in Fig 3(ii) in the frequency range of 2950 to 2820 cm^-1^. The FTIR spectra of 14-E2-14 shows the C–H stretching bands at frequencies of 2915.49 and 2847.81 cm^-1^ which are the symmetric and asymmetric C–H stretching of the methylene chain. However, in the case of the 14-E2-14+AMT mixed system, the symmetric C-H stretching band of 14-E2-14 is shifted towards higher frequencies from 2847.81 cm^-1^ (pure 14-E2-14) to 2852.82 cm^-1^ (14-E2-14+AMT mixture) along with the asymmetric C-H stretching band of 14-E2-14, which also moved to higher frequency from 2915.49 cm^-1^ to 2923.25 cm^-1^. Overall variation in frequency between pure and mixed components as a result of their interaction was not obtained too much high but found to be reproducible. The observed frequencies in the spectra of formed mixed micelles of 14-E2-14+AMT was higher than those of pure 14-E2-14 spectra. These shifts in frequency show the extent of interactions among the components of the mixed micelles formed by the 14-E2-14+AMT mixture. In general, the shifting in -CH bending and stretching frequencies reflects the interaction between the components [63].

### 3.7. UV-visible study

**Fig 4:**
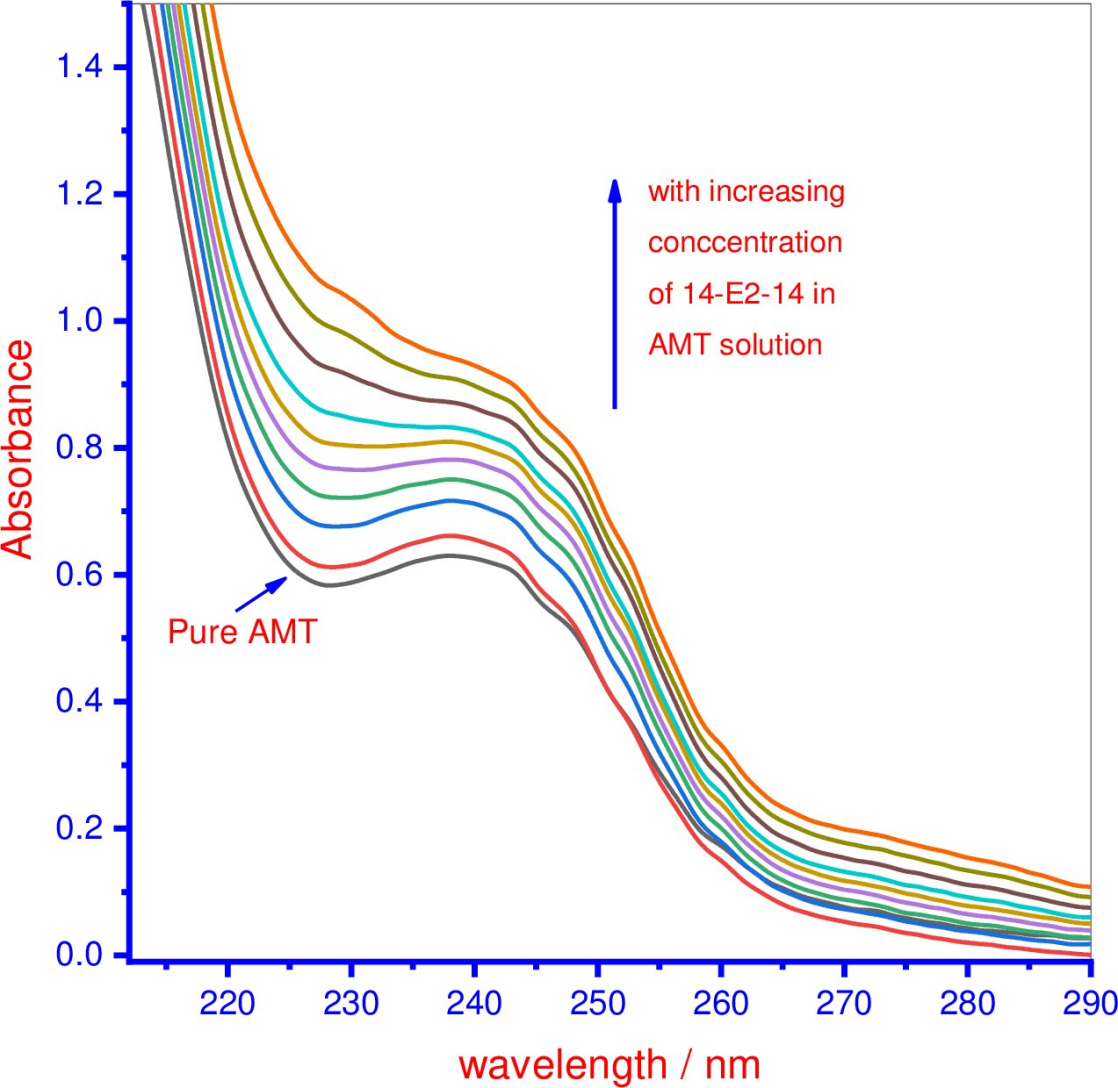
UV-visible spectra of AMT in absence and presence of increasing concentrations of 14-E2-14.

To perform UV-visible measurements, a solution of the fluorescence compound AMT (0.030 mmol.kg^-1^) was used. Titration was performed with increasing volumes of the 14-E2-14 gemini surfactant solution, placed directly into the quartz cuvette (containing 2 ml AMT). A solution of the 14-E2-14 gemini surfactant, at a fixed concentration, was prepared in a 0.030 mmol.kg^-1^ AMT solution to avoid dilution effects. UV-visible spectroscopy is a very simple, sensitive, and rapid technique. This method is also used in this study to explore the interactions amongst the components of the mixtures. The absorption spectrum of 0.030 mmol·kg^-1^ AMT solution in the aqueous system was collected and the wavelength of maximum absorbance was obtained; it fell between 238.0 and 239.0 nm. This wavelength band was attributed to a π- π* transition. A UV–vis absorption spectra of pure AMT was recorded in the absence and presence of increasing concentrations of 14-E2-14 (0.07–1.36 mmol·kg^-1^) and is given in Fig 4. All employed concentrations of 14-E2-14 are in their micellar form. However, Fig 4 shows that adding the micellar solution of 14-E2-14 to the AMT solution did not any shift significantly at all employed concentrations of 14-E2-14, and the maxima peak is the same as for AMT, but the absorption intensity of AMT rises, which is referred to as the hyperchromic effect and occurs due to the interaction between AMT and 14-E2-14. It is probably reflecting the intercalation of AMT monomers into the palisade layer of the micellar solution of 14-E2-14 [64]. The outcomes of titration revealed a clear interaction between the employed constituents as the spectral individuality of AMT vanishes as a result of an AMT–14-E2-14 complex formation [65]. The shift in maxima wavelength of AMT on adding of the 14-E2-14 solution towards lower wavelength is of low order (1 to 2 nm), hence it is somewhat challenging to reach any measurable assumption from the data.

### 3.8. Fluorescence study

#### 3.8.1. Evaluation of micellar aggregation number

**Fig 5:**
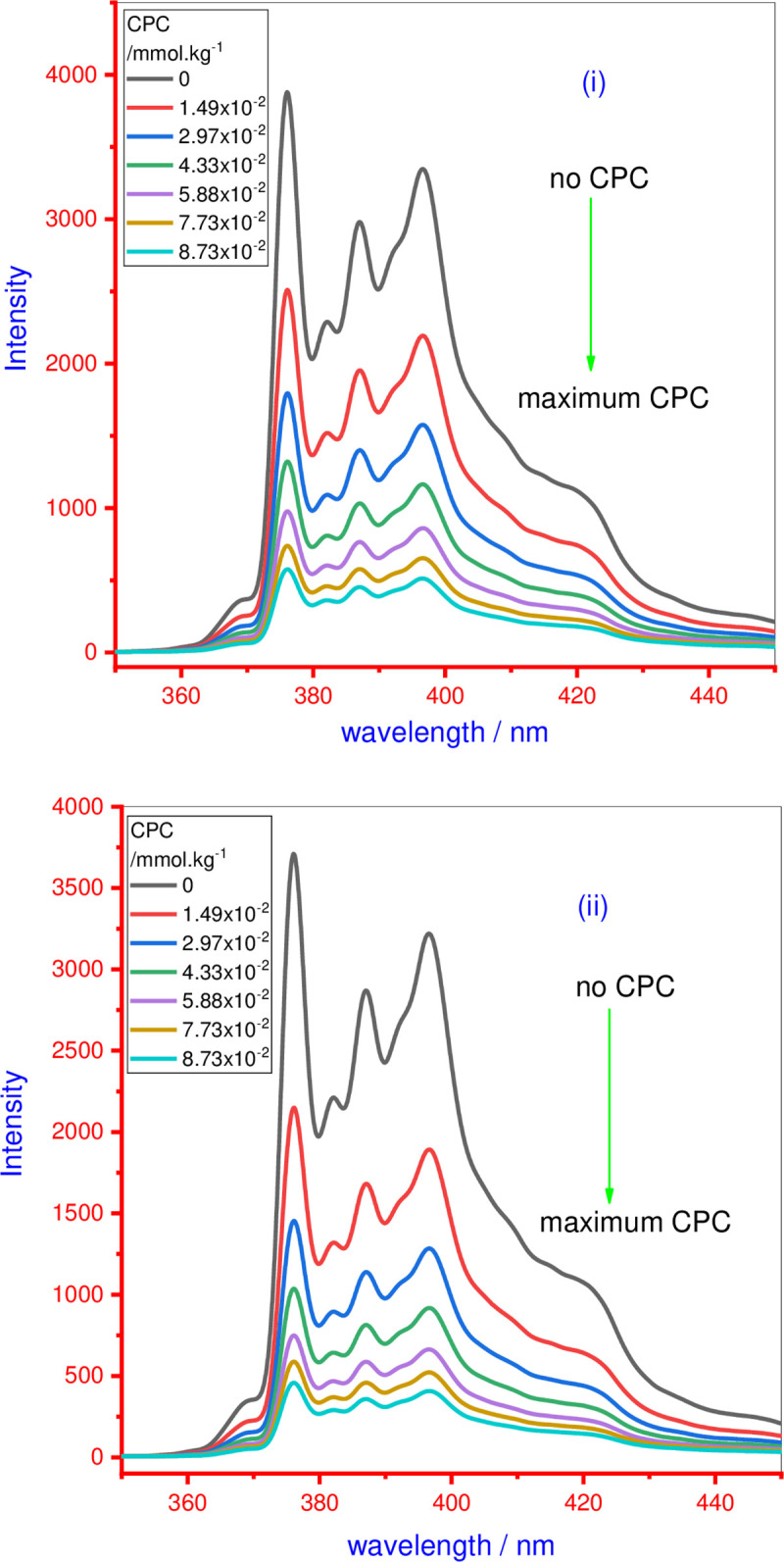
The 10^−3^ mmol.kg^-1^ pyrene (PY) solution fluorescence spectra of (i) individual 14-E2-14, and (ii) 14-E2-14 (0.8)+AMT (0.2) micellar mixed system at diverse quencher concentrations in presence of 0.050 mol.kg^-1^ NaCl.

The aggregation number *(N*_*agg*_*)* is the number of amphiphile molecules which is required for the formation of micelle. A fluorescence method, specifically steady-state fluorescence quenching, was utilized to determine the *N*_*agg*_. In the current study, PY and CPC were utilized as probe and quencher, respectively, and were obtained to be suitable to explore the *N*_*agg*_ of pure AMT, 14-E2-14 and AMT+14-E2-14 mixtures of different ratio in all solvents [66, 67]. Turro and Yekta [66] developed a method to evaluate the *N*_agg_ of micellar solution which was based on the Tachiya model [68]. The values of the aggregation number (*N*_agg_) of the current systems were determined through the following equation [66].

$$\ln\left(\frac{I_{o}}{I_{1}}\right)=\frac{N_{\mathrm{agg}}[Q]}{C_{t}‐cmc}$$ (24)

where *I*_0_ = fluorescence emission intensity of PY in the absence of the quencher and *I*_1_ = the emission intensity of PY in the presence of the quencher, [Q] = concentration of the quencher and *C*_t_ = total concentration of AMT, 14-E2-14 and AMT+14-E2-14 mixture in different ratios in the company of different solvents (water/NaCl/urea). Fig 5 shows the fluorescence emission intensity of PY in the absence and presence of increasing CPC concentration in the well above the micellar system of (i) individual 14-E2-14, and (ii) (0.8) 14-E2-14+(0.2) AMT ratio mixture in presence of 0.050 mol·kg^-1^ NaCl. Each spectrum contains five distinct emission bands ranging from between 370–400 nm. As shown in Eq (24), the relationship between [Q] and ln(*I*_0_/*I*_1_) can be used to calculate the *N*_agg_. The slope (*N*_agg_/([*C*_t_]- *cmc*)) of the straight lines are collected for all systems from plots of the ln(*I*_0_/*I*_1_) versus CPC concentration [Q]. Finally, the *N*_agg_ value was evaluated from these slopes values by putting the value of *cmc* measured by the tensiometric method and stock solution concentration used for fluorescence studies. Table 4 shows the values of *N*_agg_ of pure AMT, 14-E2-14 as well as AMT+14-E2-14 mixtures in the presence of various solvents (aqueous/0.050 mol∙kg^-1^ NaCl/0.50 and 1.0 mol∙kg^-1^ urea). In aqueous, the value of *N*_agg_ of individual AMT is found to be in good agreement with the previously stated value [7, 57, 69]. The value *N*_agg_ of singular 14-E2-14 in the aqueous system was also close to the previously reported value [41]. The *N*_agg_ value of AMT+14-E2-14 mixed systems in different ratios as well as in different solvents was found to be larger than the *N*_agg_ of the single components (Table 4). The increase in *N*_agg_ in the case of mixed systems is probably due to the formation of larger micelles (Table 4). *N*_agg_ increases with an increase in the *α*_1_ of 14-E2-14 for all the mixed surfactant systems, which can be ascribed to a decline in the micellar surface charge density which supports the incorporation of 14-E2-14 into the mixed micelle. Therefore, the synergistic mixing of AMT and 14-E2-14 retains the formed mixed micelle *N*_agg_ value in all cases, which higher than that of the single components.

**Table 4:** Aggregation number (*N*_agg_) and different associated parameters for AMT+14-E2-14 mixed system in diverse solvent at 298.15 K.

*α*_1_	*N*_agg_	*I*_1_/*I*_3_	*K*_sv_ x 10^−4^	*D*	*D*_id_
Aqueous system					
0	22	1.57	1.54	45.23	
0.2	32	1.53	16.30	41.86	27.54
0.4	40	1.38	15.63	29.66	26.79
0.6	46	1.34	10.25	26.88	27.90
0.8	57	1.32	8.09	25.36	27.63
1	28	1.33	4.90	25.74	
0.050 mol∙kg^-1^ NaCl					
0	36	1.42	1.08	33.82	
0.2	47	1.49	19.98	38.35	24.22
0.4	55	1.35	17.28	27.86	24.65
0.6	65	1.32	10.80	25.03	24.84
0.8	74	1.31	1.73	24.22	25.07
1	41	1.30	5.98	23.61	24.22
0.50 mol∙kg^-1^ urea					
0	19	1.59	1.65	46.24	
0.2	29	1.52	11.93	41.25	24.18
0.4	35	1.39	21.26	30.48	24.73
0.6	43	1.33	18.10	26.29	25.14
0.8	51	1.33	13.44	25.82	25.06
1	24	1.29	11.54	23.06	
1.0 mol∙kg^-1^ urea					
0	16	1.63	1.74	48.02	
0.2	25	1.57	8.37	45.17	25.99
0.4	31	1.41	17.55	32.12	26.47
0.6	37	1.36	15.18	28.32	27.44
0.8	44	1.33	12.53	25.92	27.27
1	19	1.32	9.10	25.05	

In the salt solution, the *N*_agg_ of single constituents as well as AMT+14-E2-14 mixtures is increased over the corresponding aqueous solution. Added NaCl in solutions of AMT, 14-E2-14, and AMT+14-E2-14 mixture, lessens the repulsions amongst the head-group of the components, causing a higher *N*_agg_ (Table 4) [70, 71]. In contrast to NaCl, in the presence of urea (both 0.50 and 1.0 mol·kg^-1^), *N*_agg_ was lower for than the aqueous solutions. The achieved *N*_agg_ values were ranked as follows: AMT, 14-E2-14, or AMT+14-E2-14 in 0.050 mol·kg^-1^ NaCl > AMT, 14-E2-14, or AMT+14-E2-14 in aqueous solution > AMT, 14-E2-14, or AMT+14-E2-14 in 0.50 mol·kg^-1^ urea> AMT, 14-E2-14, or AMT+14-E2-14 in 1.0 mol·kg^-1^ urea). Urea increases the repulsions between the head-groups of components; accordingly, *N*_agg_ decreases in urea solution. In the presence of urea, the decrease in *N*_agg_ was also mentioned earlier [72, 73]. The urea molecules are nearly 2.5 times larger than water molecules and so urea can substitute for numerous water molecules through the solvation layer of the micellar system. Overall, the *N*_agg_ value of the mixed system in 0.050 mol·kg^-1^ NaCl displayed the best synergistic behavior of AMT+14-E2-14 mixtures.

#### 3.8.2. Micropolarity (*I*_1_/*I*_3_)

The investigation of the microenvironmental properties of the micellar solutions is fascinating as not only can they make data available on the microstructure of the associated structure, but also because these properties are possibly practicably significant in several applications [74]. The ratio of the first (*I*_1_) and third (*I*_3_) vibronic peaks intensities i.e., *I*_1_/*I*_3_ called the micropolarity of the PY emission spectrum is found sensitive near the polarity index of the solubilization location of PY [75].

A small *I*_1_/*I*_3_ value (<1) specifies a nonpolar atmosphere of solubilized PY (in the hydrocarbon solvent) whereas a large value >1) indicates a polar atmosphere. The representative *I*_1_/*I*_3_ values are C_6_H_12_ = 0.6, C_6_H_5_-CH_3_ = 1.04, C_2_H_5_-OH = 1.23, CH_3_-OH = 1.33 and H_2_O = 1.84 [75]. The intensity of the fluorescence peak for individual PY is found to less, showing that PY has restricted itself in the nearness of the hydrophobic aggregate structures. Usually, the fluorescence emission spectrum of the PY probe shows five clear vibronic peaks (Fig 5) [74]. The *I*_1_ and *I*_3_ values are decreased via the increase in CPC concentration. Similarly, to determine *N*_agg_, the solution for the measurement of micropolarity (*I*_1_/*I*_3_) of all studied systems (pure AMT, 14-E2-14 and AMT+14-E2-14 mixed system in diverse ratios in all solvents) has been made well beyond the corresponding *cmc*. The measured value of *I*_1_/*I*_3_ for AMT, 14-E2-14, and AMT+14-E2-14 mixtures at different specified *α*_1_ values of 14-E2-14 in three solvents are also reported in Table 4. The ratio of *I*_1_/*I*_3_ is the measure of the polarity of the inner part of the associated structure. Typically, PY dissolves in the core of the normal micelle. The evaluated value of *I*_1_/*I*_3_ was higher than the one demonstrating that PY is mainly present or solubilized in a polar region of micelles. The values of *I*_1_/*I*_3_ in the cases of cationic AMT (1.57 in H_2_O, 1.42 in 0.050 mol·kg^-1^ NaCl, 1.59 in 0.50 mol∙kg^-1^ urea and 1.63 in 1.0 mol∙kg^-1^ urea) show that the solubilization of PY occurs in the micellar palisade layer. Whereas lower values for 14-E2-14 (1.33 in H_2_O, 1.30 in 0.050 mol·kg^-1^ NaCl, 1.29 in 0.50 mol∙kg^-1^ urea and 1.32 in 1.0 mol∙kg^-1^ urea) compared with AMT indicate slightly deeper solubilization of PY on the inner side of the palisade layer towards the core. For AMT+14-E2-14 mixed system in presence of all solvents, the *I*_1_/*I*_3_ value is determined to be decreased by the means of the increasing the *α*_1_ of 14-E2-14, once again confirming the high proportion of 14-E2-14 in mixed micelles but at highest *α*_1_ of 14-E2-14, the value of *I*_1_/*I*_3_ was found to be slightly more than or close to the *I*_1_/*I*_3_ value of pure 14-E2-14.

Table 4 also displays the *I*_1_/*I*_3_ value in the case of pure AMT, 14-E2-14 and AMT+14-E2-14 mixed system was found to be decreased in the NaCl system compared to the aqueous system, showing that in the salt system, the environment of AMT, 14-E2-14 micelles and AMT+14-E2-14 mixed micelles is less polar. However, in the urea system, the *I*_1_/*I*_3_ value for pure AMT and AMT+14-E2-14 mixed system in all ratios (except pure 14-E2-14) was higher than in the aqueous system, signifying that the environment of AMT micelles and AMT+14-E2-14 mixed micelles is more polar. With the increase of urea concentration from 0.50 to 1.0 mol·kg^-1^, the *I*_1_/*I*_3_ also increased. As is reported that in urea solvent, the head groups surface area is raised, and that producing the integration of a higher amount of water in this region of the micelle palisade layer, that enhances the polarity of the probe (PY is put away towards the exterior of the micelle to some extent that sources an additional polar atmosphere).

The Stern–Volmer equation is used here to find the equilibrium constant termed the Stern–Volmer constant (*K*_SV_) to view the interaction of the PY through the formed micelles of AMT, 14-E2-14 or mixed micelles of AMT+14-E2-14 that govern the bimolecular quenching along with unimolecular decay [76, 77]. The value of *K*_SV_ is evaluated through the following term.

$$\frac{I_{0}}{I_{1}}=1+K_{\mathrm{SV}}[Q]$$ (25)

In Eq (25), *I*_0_ and *I*_1_ are the fluorescence intensities of PY in the absence and presence of CPC, respectively. The plot of *I*_0_/*I*_1_ versus [Q] provides one type of quenching process. The variation in the intensity of the absorption of PY was not detected as the CPC is added to the PY−AMT/14-E2-14/AMT+14-E2-14 micelle and the time-resolved lifetime (*τ*) varies through the adding of CPC just as though *τ*_o_/*τ* = *I*_0_/*I*_1_ relation tracked [78]. These actualities show that the nature of quenching is dynamic. Table 4 shows all systems assessed with a *K*_SV_ value. The *K*_SV_ value rises when the solubility of the PY and CPC rises in the micellar system. The AMT+14-E2-14 mixed system in various ratios, *K*_SV_ value was found to be well above the *K*_SV_ value of singular components (AMT or 14-E2-14) except 0.8 *α*_1_ of 14-E2-14 in NaCl system, screening the higher hydrophobic environment in mixed micellization process in aqueous, NaCl, and urea (Table 4).

The apparent or experimental dielectric constant (*D*) value of AMT, 14-E2-14, and AMT+14-E2-14 mixed system in the different ratios in presence of various chosen solvent was measured through utilizing the Eq (26) [79, 80].

$$\frac{I_{1}}{I_{3}}=1.00461+0.01253D$$ (26)

In all studied systems, the *D* value was evaluated using the value of *I*_1_/*I*_3_, and these values are given in Table 4. The *D* value of AMT+14-E2-14 mixed micelles is found to be more than the *D* value of individual 14-E2-14 micelles in each solvent. However, the *D* value of pure AMT micellar solution in the presence of all solvents was different from that of the pure 14-E2-14 micelles as well as that of the AMT+14-E2-14 mixed micelles, except for 0.2 *α*_1_ of 14-E2-14 in NaCl solvent. It is clear from this data that the value of *D* is not showing any certain trend with increasing *α*_1_ of 14-E2-14. The *D* value varies between 23–48 for all studied systems. The obtained value was near to the *D* of alcohol, another indication that the environment of PY is polar.

As stated by Turro et al. [79], the ideal dielectric constant (*D*_id_) of AMT+14-E2-14 mixed system mixed micelles in the presence of all solvents is calculated from the equation below [80, 81].

$$D_{\mathrm{id}}=D_{1}X_{1}+D_{2}X_{2}$$ (27)

The calculated *D*_id_ values for all systems are listed in Table 4 and they show that the value of *D* is different from the corresponding *D*_id_ value. This again shows that the AMT+14-E2-14 mixed system contains mixed micelles with attractive interactions.

## 4. Conclusions

Although the interaction between the drug and the surfactant is necessary to improve actual drug release and delivery and avoid drug bioavailability issues, the performance of the surfactant can be significantly altered by the extent of various interactions (hydrophobic and hydrophilic) that occur both inside and outside of the cell with various additives. Before a surfactant can be developed into an appropriate drug carrier, a broad analysis must be performed to examine the association behaviors between the surfactant and the intended drug. The current study investigates the mixed micellization of AMT and a green gemini surfactant in different ratios in the presence of various solvents using tensiometric and fluorometric methods. Outcomes specify the nonideal behavior of mixed systems inspected with an attractive and synergistic interaction between components in the mixed state. In the salt or urea system, the *cmc* value of pure and mixed systems decreases or increases, respectively. A negative *β*^m^ value lower than *β*^σ^ shows less attractive interactions in mixed micelles than in the equivalent mixed monolayer. The thermodynamic data proves that the $\Delta G_{mic}^{\circ }$ of micellization is negative, demonstrating the spontaneity of the micellization, and increases gradually with increasing *α*_1_ of 14-E2-14 but the values of $\Delta G_{\mathrm{ad}}^{\circ }$ in all cases were found to be more than their respective system $\Delta G_{mic}^{\circ }$ values. The activity coefficient and mixed micellar and mixed monolayer composition data show that the micelles as well as the monolayers are primarily composed of the 14-E2-14 surfactant. A higher *N*_agg_ of the AMT+14-E2-14 mixed system in all solvents shows that micellar growth is a case of positive attractive or synergism interaction. The FT-IR spectroscopic data reflects the changes in 14-E2-14/AMT in aqueous solution. The UV-visible study results display a clear interaction between AMT and 14-E2-14 in an aqueous system. The fluorescence study of AMT through increasing *α*_1_ of 14-E2-14 indicates the presence of hydrophobic interactions between them and shows that the binding ability of both components with each other increases or decreases in salt or urea system as compared with the aqueous solution. Overall, the obtained results are significant for the expansion of efficient drug delivery models.

## References

[1] Rosen MJ. Surfactants and interfacial phenomena. 3rd ed. New York: Wiley; 2004.

[2] Ren ZH, Huang J, Zheng YC, Lai L, Hu LL. Effect of isopropanol on the micellization of binary mixture containing amino sulfonate amphoteric surfactant in aqueous solution: Mixing with octylphenol polyoxyethylene ether (7). J. Mol. Liquids 2017; 236: 101–106.

[3] Dai C, Yan Z, You Q, Du M, Zhao M. Formation of worm-like micelles in mixed n-hexadecyln-methylpyrrolidinium bromide-based cationic surfactant and anionic surfactant systems. PLoS ONE. 2014; 9: e102539 10.1371/journal.pone.0102539 25019152 PMC4097072

[4] Kumar D, Rub MA. Catalytic role of 16-s-16 micelles on condensation reaction of ninhydrin and metal-dipeptide complex. J. Phys. Org. Chem. 2019; 32: e3918.

[5] Kumar D, Rub MA, Role of cetyltrimethylammonium bromide (CTAB) surfactant micelles on kinetics of [Zn(II)-Gly-Leu]+ and ninhydrin. J. Mol. Liquids 2019; 274: 639–645.

[6] Su F, Alam R, Mei Q, Tian Y, Meldrum DR. Micelles as Delivery Vehicles for Oligofluorene for Bioimaging. PLoS ONE 2011; 6(9): e24425 10.1371/journal.pone.0024425 21915324 PMC3167853

[7] Attwood D, Florence AT. Surfactant systems, their chemistry, pharmacy and biology New York: Chapman and Hall; 1983.

[8] Rosen MJ, Tracy DJ. Gemini surfactants. J. Surf. Detergents 1998; 1: 547–554.

[9] Menger FM, Littau CA, Gemini-surfactants: synthesis properties. J. Am. Chem. Soc. 1991; 113: 1451–1452.

[10] Sharma R, Kamal A, Abdinejad M, Mahajan RK, Kraatz H.-B. Advances in the synthesis, molecular architectures and potential applications of gemini surfactants. Adv. Colloid Interface Sci. 2017; 248: 35−68. 10.1016/j.cis.2017.07.032 28800974

[11] Xia J, Zana R. Applications of gemini surfactants In gemini surfactants: Synthesis, interfacial and solution phase behavior and applications. Zana R., Xia J. (Eds.); Marcel New York: Dekker Inc.; 2004.

[12] Kim BK, Doh KO, Bae YU, Seu YB. Synthesis and optimization of cholesterol-based diquaternary ammonium gemini surfactant (Chol-GS) as a new delivery vector. J. Microbiol. Biotechnol. 2011; 21: 93−99. 10.4014/jmb.1008.08012 21301198

[13] Tehrani-Bagha AR, Oskarsson H, Ginkel, CGV. Cationic ester-containing gemini surfactants: Chemical hydrolysis and biodegradation. J. Colloid Interface Sci. 2007: 312: 444–452. 10.1016/j.jcis.2007.03.044 17481647

[14] Zhinong G, Shuxin T, Qi Z, Yu Z, Bo L, Yushu G, et al Synthesis and surface activity of biquaternary ammonium salt gemini surfactants with ester bond. Wuhan Univ. J. Natural Sci. 2008; 13: 227–231.

[15] Hamme JDV, Singh A, Ward OP. Physiological aspects. Part 1 in a series of papers devoted to surfactants in microbiology and biotechnology. Biotechnol. Adv. 2006; 24: 604–620. 10.1016/j.biotechadv.2006.08.001 16979315

[16] Fatma N, Panda M, Ansari WH, Kabir-ud-Din. Environment-friendly ester bonded gemini surfactant: Mixed micellization of 14-E2-14 with ionic and nonionic conventional surfactants. J. Mol. Liquids 2015; 211: 247–255.

[17] Mahajan S, Sharma R, Mahajan RK. Interactions of new 1,8-diazabicyclo[5.4.0]undec-7-ene (DBU) based surface active ionic liquids with amitriptyline hydrochloride: Micellization and interfacial studies. Colloids and Surfaces A: Physicochem. Eng. Aspects 2013; 424: 96–104.

[18] Alam MS, Naqvi AZ, Kabir-ud-Din. Surface and micellar properties of some amphiphilic drugs in the presence of additives. J. Chem. Eng. Data 2007; 52: 1326–1331.

[19] Khan F, Rub MA, Azum N, Asiri AM. Mixtures of antidepressant amphiphilic drug imipramine hydrochloride and anionic surfactant: Micellar and thermodynamic investigation. J. Phys. Org. Chem. 2018; 31: e3812.

[20] Mahajan RK, Mahajan S, Bhadani A, Singh, S. Physicochemical studies of pyridinium gemini surfactants with promethazine hydrochloride in aqueous solution. Phys. Chem. Chem. Phys. 2012; 14: 887–898. 10.1039/c1cp22448d 22119804

[21] Taboada P, Attwood D, Ruso JM, Garcia M, Mosquera V. Static and dynamic light scattering study on the association of some antidepressants in aqueous electrolyte solutions. Phys. Chem. Chem. Phys. 2000; 2: 5175–5179.

[22] Mahajan S, Mahajan RK. Interactions of phenothiazine drugs with surfactants: A detailed physicochemical overview. Adv. Colloid Interface Sci. 2013; 199–200: 1–14. 10.1016/j.cis.2013.06.008 23933135

[23] Bagheri A, Ahmadi SMA. Mixed micellization between amphiphilic drug propranolol hydrochloride and cetyltrimethylammonium bromide surfactant in aqueous medium. J Mol. Liquids 2017; 230: 254–260.

[24] Khan F, Sheikh MS, Rub MA, Azum N, Asiri A.M. Antidepressant drug amitriptyline hydrochloride (AMT) interaction with anionic surfactant sodium dodecyl sulfate in aqueous/brine/urea solutions at different temperatures. J. Mol. Liquids 2016; 222: 1020–1030.

[25] Zhu S., Liu L, Cheng F. Influence of spacer nature on the aggregation properties of anionic gemini surfactants in aqueous solutions. J. Surfactant Deterg. 2011; 14: 221–225.

[26] Jakubowska A. Interactions of different counterions with cationic and anionic surfactants. J. Colloid Interface Sci. 2010; 346: 398–404. 10.1016/j.jcis.2010.03.043 20381811

[27] Kuharski RA, Rossky PJ. Molecular dynamics study of solvation in urea water solution. J. Am. Chem. Soc. 1984; 106: 5786–5793.

[28] Kuharski RA, Rossky PJ. Solvation of hydrophobic species in aqueous urea solution: A molecular dynamics study. J. Am. Chem. Soc. 1984; 106: 5794–5800.

[29] Asakawa T., Hashikawa M., Amada K., Miyagishi S. Effect of urea on micelle formation of fluorocarbon surfactants. Langmuir 1995; 11: 2376–2379.

[30] Clint JH. Micellization of mixed nonionic surface active agents. J. Chem. Soc. Faraday Trans. 1975; 71: 1327–1334.

[31] Maeda H. A simple thermodynamic analysis of the stability of ionic/nonionic mixed micelles, J. Colloid Interface Sci. 1995; 172: 98–105.

[32] Rubingh DN. Mixed Micelle Solutions In: Mittal KL (Ed.), Solution chemistry of surfactants, New York: Plenum Press; 1979.

[33] Bagheri A.; Jafari-Chashmi P. Study of aggregation behavior between Nlauryl sarcosine sodium and dodecyltrimethylammonium bromide in aqueous Solution, using conductometric and spectrophotometric techniques. J. Mol. Liquids 2019; 282: 466–473.

[34] Motomura K, Aratono M. Mixed Surfactant Systems. Ogino K, Abe M (Eds.). Dekker: New York; 1998.

[35] Bagheri A, Khalili P. Synergism between non-ionic and cationic surfactants in a concentration range of mixed monolayers at an air–water interface. RSC Adv. 2017; 7: 18151–18161.

[36] Ghasemi A, Bagheri A. Effects of alkyl chain length on synergetic interaction and micelle formation between a homologous series of n-alkyltrimethylammonium bromides and amphiphilic drug propranolol hydrochloride. J. Mol. Liquids 2020; 298: 111948.

[37] Jojart B, Posa M, Fiser B, Szori M, Farkas Z, Viskolcz B. Mixed micelles of sodium cholate and sodium dodecylsulphate 1:1 binary mixture at different temperatures–experimental and theoretical investigations. PLoS ONE 2014; 9: e102114 10.1371/journal.pone.0102114 25004142 PMC4087020

[38] Zhou Q, Rosen MJ. Molecular interactions of surfactants in mixed monolayers at the air/aqueous solution interface and in mixed micelles in aqueous media: The regular solution approach. Langmuir 2003; 19: 4555–4562.

[39] Rub MA, Azum N, Asiri AM. Self-association behavior of an amphiphilic drug nortriptyline hydrochloride under the influence of inorganic salts. Russian J. Phy. Chem. B 2016; 10: 1007–1013.

[40] Saha U, Banerjee A, Das B. Drug-surfactant comicellization: Propranolol hydrochloride-surface active ionic liquid systems in aqueous medium. J. Mol. Liquids 2020; 309: 113164.

[41] Akram M, Bhat IA, Kabir-ud-Din. Effect of salt counterions on the physicochemical characteristics of novel green surfactant, ethane-1,2-diyl bis(N,N-dimethyl-N-tetradecylammoniumacetoxy) dichloride. Colloids and Surfaces A: Physicochem. Eng. Aspects 2016; 493: 32–40.

[42] Rosen MJ, Hua XY. Surface concentrations and molecular interactions in binary mixtures of surfactants. J. Colloid Interface Sci. 1982; 86: 164–172.

[43] Rub MA, Azum N, Khan F, Asiri AM. Surface, micellar, and thermodynamic properties of antidepressant drug nortriptyline hydrochloride with TX-114 in aqueous/urea solutions. J. Phys. Org. Chem. 2017; 30: e3676.

[44] Srivastava A, Uchiyama H, Wada Y, Hatanaka Y, Shirakawa Y, Kadota K, et al Mixed micelles of the antihistaminic cationic drug diphenhydramine hydrochloride with anionic and non-ionic surfactants show improved solubility, drug release and cytotoxicity of ethenzamide. J. Mol. Liquids 2019; 277: 349–359.

[45] Kumar D, Rub MA, Azum N, Asiri AM. Mixed micellization study of ibuprofen (sodium salt) and cationic surfactant (conventional as well as gemini). J. Phys. Org. Chem. 2018; 31: e3730.

[46] Azum N, Naqvi AZ, Rub MA, Asiri A.M. Multi-technique approach towards amphiphilic drug-surfactant interaction: a physicochemical study. J. Mol. Liquids 2017; 240: 189–195.

[47] Rub MA, Azum N, Asiri A.M. Interaction of cationic amphiphilic drug nortriptyline hydrochloride with TX-100 in aqueous and urea solutions and the studies of physicochemical parameters of the mixed micelles. J. Mol. Liquids 2016; 218: 595–603.

[48] Patel R, Khan AB, Dohare N, Ali MM, Rajor HK. Mixed micellization and interfacial properties of ionic liquid-type imidazolium gemini surfactant with amphiphilic drug amitriptyline hydrochloride and its thermodynamics. J. Surfact. Deterg. 2015; 18: 719–728.

[49] Yousuf S, Akram M, Kabir-ud-Din. Effect of salt additives on the aggregation behavior and morphology of 14-E2-14. Colloids and Surfaces A: Physicochem. Eng. Aspects 2014; 463: 8–17.

[50] Hiren P, Gaurav R., Heiko, H. Effects of glycerol and urea on micellization, membrane partitioning and solubilization by a non-ionic surfactant. Biophysical Chemistry 2010; 150: 119–128. 10.1016/j.bpc.2010.03.015 20417021

[51] Mukherjee P. The nature of the association equilibria and hydrophobic bonding in aqueous solutions of association colloids. Adv. Colloid Interface Sci. 1967; 1: 242–275.

[52] Kumar D, Azum N, Rub MA, Asiri AM. Aggregation behavior of sodium salt of ibuprofen with conventional and gemini surfactant. J. Mol. Liq. 2018; 262: 86–96.

[53] Sulthana SB, Rao PVC, Bhat SGT, Rakshit AK. Interfacial and thermodynamic properties of SDBS-C12E10 mixed micelles in aqueous media: effect of additives. J. Phys. Chem. B 1998; 102: 9653–9660.

[54] Kumar D, Rub MA. Aggregation behavior of amphiphilic drug promazine hydrochloride and sodium dodecylbenzenesulfonate mixtures under the influence of NaCl/urea at various concentration and temperatures. J. Phys. Org. Chem. 2016; 29: 394–405.

[55] Kuamr D, Rub MA. Effect of anionic surfactant and temperature on micellization behavior of promethazine hydrochloride drug in absence and presence of urea. J. Mol. Liquids 2017; 238: 389–396.

[56] Kumar D, Hidayathulla S, Rub MA. Association behavior of a mixed system of the antidepressant drug imipramine hydrochloride and dioctyl sulfosuccinate sodium salt: Effect of temperature and salt. J. Mol. Liquids 2018; 271: 254–264.

[57] Rub MA. Aggregation and interfacial phenomenon of amphiphilic drug under the influence of pharmaceutical excipients (green/biocompatible gemini surfactant). PLoS ONE 2019; 14: e0211077 10.1371/journal.pone.0211077 30726255 PMC6364909

[58] Sugihara G, Miyazono AM, Nagadome S, Oida T, Hayashi Y, Ko JS. Adsorption and micelle formation of mixed surfactant systems in water II: A combination of cationic gemini-type surfactant with MEGA-10. J. Oleo Sci. 2003; 52: 449–461.

[59] Tanford C. The hydrophobic effect: formation of micelles and biological membranes New York: Wiley; 1980.

[60] Myers D. Surfactant Science and Technology. 3rd ed. Wiley Intersciences, John Wiley & Sons: New Jersey; 2006.

[61] Padalkar KV, Gaikar VG, Aswal VK. Characterization of mixed micelles of sodium cumene sulfonate with sodium dodecyl sulfate and cetyl trimethylammonium bromide by SANS, FT-IR spectroscopy and NMR spectroscopy. J. Mol. Liquids 2009; 144: 40–49.

[62] Gaikar VG, Padalkar KV, Aswal VK. Characterization of mixed micelles of structural isomers of sodium butyl benzene sulfonate and sodium dodecyl sulfate by SANS, FTIR spectroscopy and NMR spectroscopy. J. Mol. Liquids 2008; 138: 155–167.

[63] Kumar H, Katal A. Interaction of cationic surfactant cetyltrimethylammonium bromide (CTAB) with hydrophilic ionic liquid 1 butyl 3 methylimidazolium chloride [C4mim][Cl] at different temperatures–Conductometric and FTIR spectroscopic study. J. Mol. Liquids 2018; 266: 252–258.

[64] Akram M, Bhat IA, Berekute AK, Kabir-ud-Din. Solution behaviour of an ester-functionalized gemini surfactant, ethane-1,2-diyl bis(N,N-dimethyl-N-dodecylammoniumacetoxy) dichloride in the presence of inorganic and organic salts. J. Industrial Eng. Chem. 2016; 40: 161–167.

[65] Mahajan S, Mahajan RK. Interactions of phenothiazine drugs with bile salts: Micellization and binding studies. J. Colloid Interface Sci. 2012; 387: 194–204. 10.1016/j.jcis.2012.07.085 22939256

[66] Turro NJ, Yekta A. Luminescent probes for detergent solutions. A simple procedure for determination of the mean aggregation number of micelles. J. Am. Chem. Soc. 1978; 100: 5951–5952.

[67] Rub MA, Azum N, Khan F, Asiri AM. Aggregation of sodium salt of ibuprofen and sodium taurocholate mixture in different media: A tensiometry and fluorometry study. J. Chem. Thermodynamics 2018; 121: 199–210.

[68] Tachiya M. Application of a generating function to reaction kinetics in micelles. Kinetics of quenching of luminescent probes in micelles. Chem. Phys. Lett. 1975; 33: 289–292.

[69] Rub MA, Azum N, Asiri AM, Alfaifi SYM, Alharthi SS. Interaction between antidepressant drug and anionic surfactant in low concentration range in aqueous/salt/urea solution: A conductometric and fluorometric study. J. Mol. Liquids 2017; 227: 1−14.

[70] Dar AA, Rather GM, Ghosh S, Das A.R. Micellization and interfacial behavior of binary and ternary mixtures of model cationic and nonionic surfactants in aqueous NaCl medium. J. Colloid Interface Sci. 2008; 322: 572–581. 10.1016/j.jcis.2008.03.022 18439614

[71] Azum N, Ahmed A, Rub MA, Asiri AM, Alamery SF. Investigation of aggregation behavior of ibuprofen sodium drug under the influence of gelatin protein and salt. J. Mol. Liq. 2019; 290: 111187.

[72] Costantino L, D’Errico G, Roscigno P, Vitagliano V. Effect of urea and alkylureas on micelle formation by a nonionic surfactant with short hydrophobic tail at 25°C. J. Phys. Chem. B 2000; 104: 7326–7333.

[73] Azum N, Rub MA, Asiri AM. Self-association and micro-environmental properties of sodium salt of ibuprofen with BRIJ-56 under the influence of aqueous/urea solution. J. Disp. Sci. Tech. 2017; 38: 96–104.

[74] Hierrezuelo JM, Aguiar J, Ruiz CC, Stability, interaction, size, and microenvironmental properties of mixed micelles of decanoyl-N-methylglucamide and sodium sodecyl sulfate. Langmuir 2004; 20: 10419–10426. 10.1021/la048278i 15544368

[75] Kalyanasundaran K, Thomas JK. Environmental effects on vibronic band intensities in pyrene monomer fluorescence and their application in studies of micellar systems. J. Am. Chem. Soc. 1977; 99: 2039–2044.

[76] Das S, Naskar B, Ghosh S. Influence of temperature and organic solvents (isopropanol and 1,4-dioxane) on the micellization of cationic gemini surfactant (14-4-14). Soft Matter 2014; 10: 2863–2875. 10.1039/c3sm52938j 24668039

[77] Rub MA, Khan JM, Azum N, Asiri A.M. Influence of antidepressant clomipramine hydrochloride drug on human serum albumin: Spectroscopic study. J. Mol. Liquids 2017; 241: 91–98.

[78] Lakowicz JR. Principles of fluorescence spectroscopy. New York: Plenum;1999.

[79] Turro NJ, Kuo PL, Somasundaran P, Wong K. Surface and bulk interactions of ionic and nonionic surfactants. J. Phys. Chem. 1986; 90: 288–291.

[80] Rub MA, Azum N, Asiri AM. Binary mixtures of sodium salt of ibuprofen and selected bile salts: interface, micellar, thermodynamic, and spectroscopic study. J. Chem. Eng. Data 2017; 62: 3216–3228.

[81] Rub MA, Khan F, Sheikh MS, Azum N, Asiri AM. Tensiometric, fluorescence and ^1^H NMR study of mixed micellization of non-steroidal anti-inflammatory drug sodium salt of ibuprofen in the presence of non-ionic surfactant in aqueous/urea solutions. J. Chem. Thermodyn. 2016; 96: 196–207.

